# Effects of Whole-Body, Local, and Modality-Specific Vibration Therapy on Gait in Parkinson’s Disease: A Systematic Review and Meta-Analysis

**DOI:** 10.3390/biomedicines13102505

**Published:** 2025-10-14

**Authors:** Ji-Woo Seok, Se-Ra Park

**Affiliations:** 1 Digital Health Research Division, Korea Institute of Oriental Medicine, Daejeon 34054, Republic of Korea; suk6124@kiom.re.kr; 2 Korean Medicine (KM) Convergence Research Division, Korea Institute of Oriental Medicine, Daejeon 34054, Republic of Korea

**Keywords:** Parkinson’s disease, gait, vibration therapy, meta-analysis

## Abstract

**Background/Objectives:** Gait dysfunction is a major contributor to disability and reduced quality of life in Parkinson’s disease (PD). Although pharmacological treatments and exercise-based rehabilitation programs provide partial improvement, residual gait dysfunction often persists. Given these limitations, there has been growing interest in non-pharmacological and non-invasive strategies such as vibration therapy (VT). However, previous systematic reviews and meta-analyses have yielded inconsistent findings, largely summarizing the presence or absence of treatment effects without clarifying the clinical or therapeutic conditions under which VT may be most effective. Therefore, this study aimed to systematically review and synthesize evidence on the efficacy of VT for improving gait in PD and to identify clinical and therapeutic factors influencing treatment outcomes. **Methods:** A systematic search of PubMed, Web of Science, Embase, and the Cochrane Library was conducted with no restrictions on the search period, including studies published up to July 2025. Eligible studies included randomized and quasi-experimental clinical trials that evaluated the effects of VT on gait-related outcomes in patients with Parkinson’s disease. Data extraction followed the PRISMA 2020 guidelines, and the risk of bias was assessed using the Cochrane RoB 2 and ROBINS-I tools. Multilevel random-effects meta-analyses were conducted to estimate pooled effect sizes for gait outcomes, and meta-regression and subgroup analyses were performed based on disease stage, medication status, and vibration parameters. **Results:** A total of 14 studies (11 randomized and 3 non-randomized) were included. The pooled analysis showed that VT significantly improved gait performance in PD (Hedges’ g = 0.270, 95% CI: 0.115–0.424; 95% PI: −0.166–0.705). The sensitivity analysis restricted to randomized controlled trials yielded a comparable significant effect (g = 0.316, 95% CI: 0.004–0.628). Greater benefits were observed in patients with higher clinical severity, while the moderating effect of levodopa dosage was not significant. Optimal effects were identified with frequencies of 51–100 Hz, session durations ≤3 min, and 2–3 sessions per week. Improvements were evident in gait speed, cycle, and magnitude, whereas no consistent effects were observed for freezing of gait or gait variability. **Conclusions:** VT yields small but statistically significant improvements in fundamental gait parameters in Parkinson’s disease, particularly under optimized stimulation conditions and in individuals with greater disease severity. Although the pooled effect was modest and the certainty of evidence was rated as very low according to GRADE, these findings cautiously support the potential of vibration-based interventions as a supportive, non-pharmacological, and non-invasive adjunct within broader rehabilitation programs, rather than as a stand-alone treatment.

## 1. Introduction

Parkinson’s disease (PD) is a progressive neurodegenerative disorder in which clinical symptoms inevitably worsen over time, necessitating long-term management and multidisciplinary care [1,2]. With the ongoing demographic shift toward an aging global population, its prevalence continues to rise, posing not only a major medical challenge but also a substantial socioeconomic and public health burden [3,4]. Clinically, PD is defined by its cardinal motor manifestations, including bradykinesia, rigidity, tremor, and postural instability [5]. Among these, gait disturbance emerges as one of the most disabling features. Impairments in gait severely compromise patients’ independence and quality of life, while markedly increasing the risk of falls, injuries, and loss of autonomy.

To address gait impairments in PD, pharmacological and rehabilitative strategies have been widely employed to address gait dysfunction, yet each presents important limitations. Levodopa, while initially effective, exhibits diminishing returns over time and is frequently associated with motor fluctuations and dyskinesias with prolonged use, thereby limiting its long-term utility for gait management [6,7]. Similarly, physiotherapy and structured exercise programs have demonstrated benefits for motor function, but their sustained efficacy is often undermined by poor adherence in advanced stages, with studies reporting adherence rates ranging from approximately 79% in home-based programs to as low as 60% in tele-exercise interventions [8,9]. Moreover, not only does low adherence erode therapeutic gains, but poor attendance rates and high dropout rates further compromise the longevity of treatment effects [10]. Taken together, these limitations underscore that pharmacological and physiotherapeutic interventions alone are insufficient for maintaining gait function over the disease course. Accordingly, there is a need for adjunctive, non-pharmacological treatment strategies, and various alternative approaches have begun to receive growing attention as potential options to address this need.

Given these limitations, there has been growing interest in adjunctive, non-pharmacological, and non-invasive strategies that directly target gait dysfunction through alternative mechanisms. Among these, VT has emerged as a particularly promising therapeutic option. Mechanistically, VT is proposed to stimulate proprioceptive receptors within muscles and joints, such as muscle spindles and Golgi tendon organs, thereby augmenting afferent feedback related to body position and movement, which in turn enhances the regulation of muscle tone [11]. These sensory inputs are subsequently conveyed to the spinal cord and supraspinal centers, where they promote sensorimotor integration and improve the precision and timing of motor execution [12,13]. With repeated application, vibratory stimulation can further reorganize neuromuscular activation patterns by reducing excessive co-contraction and facilitating more efficient recruitment of agonist muscles [14]. Collectively, these mechanisms suggest that VT may enhance both the amplitude and rhythmicity of gait. Notably, because vibration-based interventions are largely passive and require minimal voluntary effort, they may be particularly well suited for individuals with advanced Parkinson’s disease who experience pronounced rigidity or restricted mobility [15,16].

Nevertheless, clinical studies investigating VT in PD have reported mixed findings. Reported outcomes vary considerably, likely due to heterogeneity in vibration parameters (e.g., frequency, amplitude, duration, and intervention length), patient characteristics (e.g., disease stage, medication use), and methodological design. Previous meta-analyses have highlighted these inconsistencies, noting limitations such as small sample sizes, short follow-up periods, and substantial variation in intervention protocols [17,18,19]. Consequently, the optimal parameters and clinical conditions under which VT may exert the greatest benefit remain unclear.

Previous systematic reviews and meta-analyses have reported inconsistent findings and provided only limited levels of evidence. For example, Dincher et al. [17] focused narrowly on harmonic versus randomized WBV, but the small number of included trials precluded firm conclusions. Fischer et al. [18] synthesized the long-term effects of WBV across heterogeneous populations, such as older adults and stroke patients, yet did not clarify how intervention characteristics or patient-related factors shaped treatment outcomes. Zhao et al. [20] restricted their analysis to Parkinson’s disease and reported improvements only in selected measures (e.g., UPDRS-III, TUG), while failing to differentiate effects across vibration parameters or clinical subgroups. Collectively, these prior reviews largely summarized the presence or absence of therapeutic effects without adequately addressing the methodological diversity, limited sample sizes, or the critical question of under which conditions VT exerts its greatest benefits.

Against this background, the present study aims to provide a comprehensive evaluation of the effects of VT on gait in patients with Parkinson’s disease through a systematic review and meta-analysis. The outcomes examined include not only fundamental gait parameters such as gait speed, step length, stride cycle, and movement amplitude, but also functional assessments such as the Timed Up and Go (TUG) test and the 6-Minute Walk Test (6 MWT). Rather than merely confirming the overall efficacy of VT, this study seeks to elucidate how clinical and therapeutic factors such as disease stage, levodopa dosage, vibration frequency, amplitude, session number, total exposure time, and control type moderate treatment effects. Furthermore, by disentangling differential effects across specific gait domains, this work aims to generate more precise and clinically meaningful evidence to inform the development of tailored rehabilitation strategies for individuals with PD.

## 2. Methods

### 2.1. Study Design

This systematic review and meta-analysis were conducted to evaluate the effects of VT on gait-related outcomes in patients with Parkinson’s disease. The study protocol was prospectively registered in the International Prospective Register of Systematic Reviews (PROSPERO; registration number: CRD420251114948, https://www.crd.york.ac.uk/PROSPERO/view/CRD420251114948, accessed on 25 August 2025), and the review was conducted in accordance with the registered protocol. The review adhered to the PRISMA 2020 guidelines, and the corresponding PRISMA 2020 checklist is provided in Supplementary File S1. The study design, the literature search and selection, data extraction, risk of bias assessment, and data synthesis procedures were all implemented based on a predefined protocol [21,22,23].

### 2.2. Literature Search and Screening

A systematic literature search was conducted in five electronic databases: PubMed, the Cochrane Central Register of Controlled Trials (CENTRAL), Embase via Ovid, MEDLINE, and the Science Citation Index (Web of Science). The search period had no lower limit, and studies published up to 31 July 2025 were included. The search was limited to articles published in English. The detailed search strategy, including full Boolean syntax for each database, is available in the PROSPERO registration record (CRD420251114948).

The search strategy was developed based on the PICO framework, with “Parkinson’s disease” OR “Parkinson disease” OR “PD” as the population, “vibration therapy” OR “whole body vibration” OR “focal muscle vibration” OR “mechanical vibration” OR “stochastic resonance vibration” as the intervention, “placebo” OR “sham” OR “no treatment” OR “usual care” OR “treatment as usual” OR “TAU” OR “active control” OR “physical rehabilitation” as the comparison, and “gait” OR “walking” OR “locomotion” OR “gait speed” OR “stride length” OR “step length” OR “cadence” OR “Timed Up and Go” OR “TUG” OR “MWT” OR “freezing of gait” as the outcome. These terms were combined using Boolean operators (AND, OR) and applied to the title, abstract, and keyword fields.

Two reviewers independently screened the titles and abstracts of the identified records to determine potential eligibility, followed by full-text assessment to confirm inclusion. To minimize selection bias, the screening process was performed independently by two reviewers, with disagreements resolved by consensus with a third reviewer. Reference lists and citations of the included studies were also examined to identify additional eligible studies, and citation tracking (snowballing) and expert consultation were performed to capture potentially unpublished or overlooked studies. Because only English-language and published articles were included, the potential for language and publication bias was acknowledged and further evaluated through funnel-plot asymmetry and statistical tests.

### 2.3. Eligibility Criteria

This study included research that met specific criteria for participants, interventions, comparators, and study designs. Participants were required to be adults aged 18 years or older with a Parkinson’s disease diagnosis, regardless of their disease stage or severity. They also had to experience gait disturbance or limited mobility. Gender was not a restriction for inclusion. However, studies were excluded if they involved participants with other neurological or musculoskeletal conditions (e.g., stroke, multiple sclerosis, spinal cord injury), or if they were conducted in pediatric populations or animal models. We also excluded studies that did not report outcomes specifically for patients with Parkinson’s disease.

Interventions were limited to clinically applicable forms of vibration therapy, such as whole-body vibration, localized vibration, or stochastic resonance vibration, whether delivered as the primary or an adjunctive intervention. Studies were excluded if they used vibration for non-therapeutic purposes, had unclear vibration protocols, or if the effects of vibration therapy could not be isolated within a complex intervention.

Eligible comparator groups included placebo or sham vibration, usual care (e.g., gentle ambulation, gentle stretching, standard medication), no intervention, or active control interventions such as physical rehabilitation. We excluded any studies that lacked a comparison group or whose interventions were unrelated to gait or motor function.

Eligible study designs included randomized and quasi-experimental studies with a control group. All included studies were required to be published in peer-reviewed journals. Excluded designs comprised case reports, case series, opinion pieces, editorials, conference abstracts without full data, single-arm before-and-after studies without a control group, and existing systematic reviews or meta-analyses. However, the reference lists of such reviews were still screened to identify potential primary studies.

### 2.4. Data Extraction

Data extraction was performed independently by at least two researchers, with a third investigator contributing to the consensus process in case of disagreement. The following information was collected from each included study using a standardized data extraction form: author name and year of publication, study design type, participant characteristics (e.g., mean age, sex, disease duration, Hoehn and Yahr stage, L-dopa dosage), sample size, intervention and comparator protocols, frequency and duration of vibration therapy, vibration therapy parameters (e.g., number of bouts, duration of each session, rest time, frequency, amplitude), application posture and type of vibration (e.g., whole-body vibration, localized vibration), type of equipment used, and outcome measures (e.g., walking speed, stride length, step width, cadence, Timed Up and Go, 6 min walk test, frequency and duration of freezing of gait) (Table 1). To standardize the reporting of vibration therapy protocols, we extracted key parameters based on the “Body Vibration Big Five” framework [24]. These five essential components included vibration frequency, amplitude, application method, session duration and frequency, and the total intervention period.

For information not available in the published articles, the original authors were contacted. All data were systematically recorded using a predefined form, and the extracted information was used for meta-analysis and qualitative analysis. Data presented visually, such as in graphs, were converted into numerical values using WebPlotDigitizer software version 5.2. When crucial statistical metrics like the mean or standard deviation were not explicitly stated, we reached out to the primary authors for the necessary information. To maintain rigor, data were extracted by two researchers working independently, and any discrepancies in data interpretation or figures were resolved by a third reviewer.

### 2.5. Assessment of Methodological Quality

The risk of bias in included randomized parallel-design studies was assessed using the Cochrane Collaboration’s Risk of Bias 2 (RoB 2) tool for parallel designs [25,26]. For randomized crossover studies, the version of the RoB 2 tool specifically designed for crossover trials was used. Non-randomized studies were assessed with the Risk of Bias in Non-randomized Studies of Interventions (ROBINS-I) tool.

All risk of bias assessments were performed independently by at least two investigators. In case of disagreements, a third investigator was included to resolve them through discussion and consensus. The assessments were conducted according to predefined criteria, including the randomization process, potential carryover effects in crossover studies, intervention implementation, handling of missing data, methods of outcome measurement, and selective reporting. If the study report had insufficient or unclear information, the original authors were contacted for additional details. The final evaluation results were used to judge the methodological quality of the included studies and to consider the level of evidence when interpreting the meta-analysis results.

### 2.6. Statistical Analysis

All meta-analyses were conducted using JASP 0.95. Effect sizes were calculated using the standardized mean difference (SMD; Hedges’ g) and 95% confidence intervals to integrate results from different scales. SMD calculations were performed according to the Cochrane Handbook recommendations [23]. If each study directly reported the mean pre- to post-test change and the standard deviation (SD) of the change, the SMD was calculated using the reported change statistics. If only the pre- and post-test group means and SDs were reported, the mean change (post-test minus pre-test) for each group was first calculated. Then, the difference in change between the experimental and control groups was standardized by the pooled SD to calculate the SMD [27,28]. For studies that did not report the SD of the change, the variance of change was imputed assuming a pre–post correlation of r = 0.50 [29]. To correct for small-sample bias, Hedges’ small-sample correction was applied [30], and the standard error (SE) of each effect size was derived from group sample sizes and effect sizes [31].

To address non-independence among multiple effect sizes from the same study, all pooled analyses were conducted using a three-level random-effects model with nested random intercepts for study and for effect sizes within studies, estimated via restricted maximum likelihood (REML) (Level 1: sampling error; Level 2: within-study effect size; Level 3: study/cluster) [32]. Residual heterogeneity was evaluated using the Q_e_ test, and the pooled effect was reported with its 95% confidence interval and 95% prediction interval. Within the multilevel model, we estimated and reported the variance components at the study level and at the within-study (effect-size) level with their 95% confidence intervals, and we assessed the necessity of each random-effects component/level using likelihood-ratio inclusion tests [32].

Univariate (not multivariate) multilevel meta-regression was conducted only for the two clinically important continuous moderators: Hoehn and Yahr stage and mean L-dopa dose. For each regression, we reported the regression coefficient (β), robust standard error (SE), test statistic, *p*-value, and the proportion of heterogeneity explained (R^2^) [33].

Other study and intervention characteristics (e.g., vibration amplitude, vibration frequency category, control group type, experimental design, intervention period, weekly frequency, total exposure time, total number of sessions, and modality) were examined only through exploratory subgroup analyses, given the limited number of studies per category and the risk of overfitting and reduced power in multivariable models. For each category level, we estimated the pooled effect size (SMD with 95% CI and 95% PI) and residual heterogeneity within levels (Q_e_ and *p*-value), while overall differences between categories were assessed using omnibus tests (Q_m_, df, *p*-value). All such subgroup findings are interpreted cautiously as exploratory and hypothesis-generating.

Publication bias was assessed visually using funnel plots in analyses with 10 or more studies and statistically using Egger’s regression and Begg’s rank correlation tests [34]. Sensitivity analysis was performed using a leave-one-out approach, in which each study was sequentially excluded and the meta-analysis was re-run [35]. Studies exerting substantial influence were identified using Baujat plots [36]. In addition, potential publication bias was further examined using Rosenthal’s fail-safe N method to estimate the number of unpublished null studies required to nullify the observed effect [37]. Duval and Tweedie’s trim-and-fill procedure was also applied to adjust for potential missing studies and to obtain an adjusted pooled estimate [38].

### 2.7. Certainty of Evidence

The certainty of evidence for each outcome was assessed using the Grading of Recommendations, Assessment, Development, and Evaluations (GRADE) approach, with ratings categorized as high, moderate, low, or very low. This rigorous assessment, independently conducted by two reviewers (with disagreements resolved through discussion), followed GRADE recommendations by considering five key domains for downgrading: risk of bias, inconsistency, indirectness, imprecision, and publication bias. Specifically, Randomized trials (RCTs and RCOs) began with a high certainty rating, while non-randomized studies (non-RCTs) started with a low certainty rating. Upgrading was possible if a clinically meaningful large effect was observed. For interpreting clinical significance, we considered not only statistical significance but also the magnitude of the effect size (Hedges’ g) and the precision of the 95% confidence interval (CI). Results were deemed clinically meaningful when the CI consistently did not cross the zero effect line, and clinical implications were judged to be present when effects were consistently observed across multiple studies. Final certainty ratings were summarized using the GRADEpro Guideline Development Tool (GRADEpro GDT, McMaster University and Evidence Prime, Hamilton, ON, Canada; https://www.gradepro.org/).

## 3. Results

### 3.1. Selection of Studies

**Figure 1:**
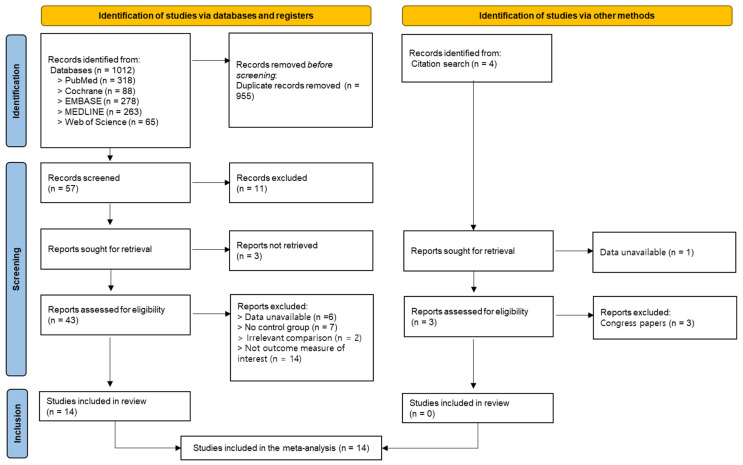
Flowchart of study selection.

A total of 1012 articles were identified through database searches: 318 from PubMed, 88 from Cochrane, 278 from EMBASE, 263 from MEDLINE, and 65 from Web of Science. After removing 955 duplicates, 57 articles remained for screening. Eleven articles were excluded after reviewing the titles and abstracts [39,40,41,42,43,44,45,46,47,48,49], and the full texts of three articles were unavailable [50,51,52]. This left 43 articles for the eligibility assessment. Of these, 29 were excluded due to unavailable data (*n* = 6) [53,54,55,56,57], absence of a control group (*n* = 7) [58,59,60,61,62,63,64], inappropriate comparisons (*n* = 2) [65,66], or outcomes not of interest (*n* = 14) [67,68,69,70,71,72,73,74,75,76,77,78,79,80]. Four additional articles were identified through citation searches; however, one was excluded due to unavailable data [81], and three were excluded because they were conference papers only [82,83,84]. Ultimately, 14 studies were included in this systematic review and meta-analysis (Figure 1) [15,16,59,78,85,86,87,88,89,90,91,92,93,94].

### 3.2. Characteristics of the Studies

A total of 14 studies were included in this meta-analysis. The study designs were diverse, comprising seven randomized controlled trials (RCTs), four randomized crossover trials, one within-subjects comparison study, one small double-blind pilot study, and one study that did not clearly specify the design but was reported as double-blind (Table 2).

All participants had Parkinson’s disease, with a mean age ranging from the late 60s to early 70s. The disease duration was approximately 5 to 12 years, and the average Hoehn and Yahr stage ranged from 2.4 to 3.1, indicating that most patients had mild to moderate disease. Some studies also reported an average daily levodopa dose, which ranged from 269 mg to 1035 mg.

The interventions were broadly categorized into whole-body vibration (WBV) and focal muscle vibration (FMV). WBV was delivered using a vibration plate (e.g., Galileo^®^, SRT Zeptor^®^, Fit Massage) while participants were standing (semi-squat or knee flexion), sitting, or performing task-based movements. Six studies applied this approach. FMV was administered using a small wearable device such as the Equistasi^®^ or an eccentric mass-based vibration device attached to a specific body part (e.g., lower extremity muscles, ankles, or lower back). A total of eight studies employed this approach.

Intervention protocols varied considerably. The intervention period ranged from a single exposure to 12 weeks of repeated sessions, and the session frequency ranged from once per week to up to 10 sessions per week. Each session typically consisted of 1–8 bouts lasting 1–3 min. The vibration frequency of WBV ranged from 6 to 25 Hz with an amplitude of 3–14 mm, whereas FMV primarily involved high-frequency, low-amplitude stimulation in the 70–100 Hz range.

Outcome measures focused on clinical and objective indicators related to gait performance, variability, and freezing of gait (FOG). Gait speed-related measures were reported in 12 studies, including cadence (5 studies), velocity or stride velocity (10 studies), stride time (1 study), the 10-Meter Walk Test (1 study), the 8-Meter Walk Test (1 study), stand-walk-sit time (1 study), walking distance (1 study), trunk velocity (step–walk–turn task) (1 study), and gait-turn time (1 study). Gait cycle-related measures were used in nine studies, including double support time (3 studies), single support time (1 study), stance duration (4 studies), and swing duration (1 study). Gait magnitude-related measures were applied in 10 studies, including step length (4 studies), stride length (3 studies), stride (2 studies), step amplitude (1 study), and mean stride length of three strides before a freeze (2 studies). Measures of gait variability were reported in four studies, including stride coefficient of variation (CV) (1 study), stance CV (1 study), swing CV (1 study), double support CV (1 study), stride velocity CV (2 studies), stride length CV (2 studies), CV of three strides before a freeze (2 studies), and stride-time CV (1 study). Task-specific measures were reported in one study, including ground reaction force (1 study), and the number of steps required during turning in the step-walk-turn task (1 study). Finally, five studies reported measures related to freezing of gait. Of these, three used the gait item of the UPDRS Part III, two used the freezing item of the UPDRS Part III, two assessed the occurrence of FOG, one assessed freezing episodes, and one assessed freezing duration.

In summary, the included studies assessed gait performance, gait cycle and amplitude, gait variability, task-specific performance, and freezing-related outcomes in patients with mild to moderate Parkinson’s disease using WBV and FMV. The types and frequencies of outcome measures varied considerably across studies, reflecting substantial methodological heterogeneity.

### 3.3. Quality Assessment Results

**Figure 2:**
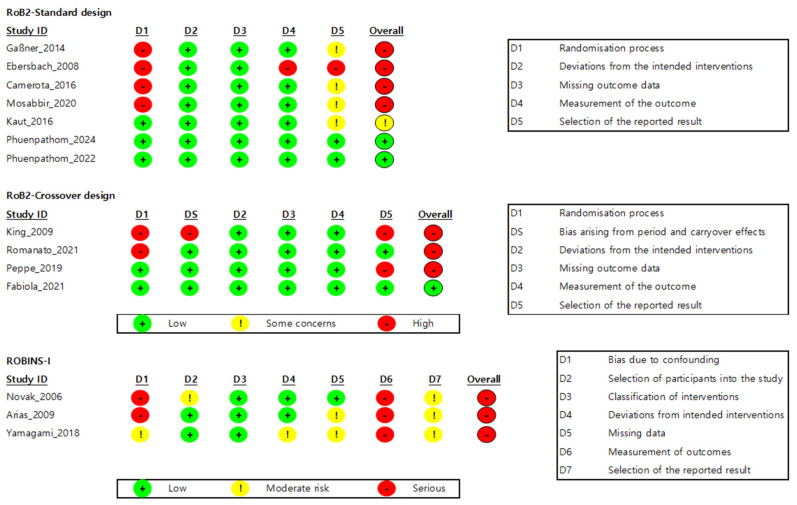
Quality assessment results by Risk of Bias. Note. Data were derived from Gaßner et al. [87], Ebersbach et al. [86], King et al. [88], Camerota et al. [15], Mosabbir et al. [89], Novak et al. [16], Kaut et al. [78], Romanato et al. [92], Peppe et al. [59], Phuenpathom et al. [90,91], Yamagami et al. [94], Fabiola et al. [93], and Arias et al. [85].

A total of 11 randomized controlled trials (RCTs) were assessed for risk of bias using Cochrane’s RoB 2.0 tool. Of these, four had a cross-over design and seven had a parallel design. As shown in Figure 2, the overall risk of bias was assessed as “low” in three studies (approximately 27.3%), “some concern” in one study (approximately 9.1%), and “high” in seven studies (approximately 63.6%). In other words, approximately 72.7% of the included RCTs had a risk of bias of “some concern” or higher in one or more domains. More specifically, many of the procedures related to randomization (Domain 1) were insufficiently described or not reported, resulting in assessments of high risk or some concern in this domain. Furthermore, one crossover trial was judged to be at high overall risk in Domain S (period and carryover effects), as the washout period was not adequately reported, and carryover effects were not statistically controlled. Finally, in Domain 5 (selection of the reported result), preregistered study protocols were rarely available, making it difficult to rule out the possibility of selective outcome reporting (Figure 2).

Meanwhile, three non-randomized studies were assessed using the ROBINS-I tool. All three studies were assessed as having an overall risk of bias of “serious”, reflecting a lack of control for key confounders and the potential for subjective judgment in the intervention exposure and outcome measurement processes. Furthermore, the absence of preregistered protocols and limited transparency in the analytic procedures were interpreted as additional factors contributing to the increased risk of bias (Figure 2).

### 3.4. Effect of Vibration Intervention on Gait in Parkinson’s Disease

To address the dependence of multiple effect sizes within studies, we used a three-level random-effects model in which effect sizes were nested within studies. A total of 71 effect sizes were derived from 14 studies (clusters). The pooled effect was a standardized mean difference (SMD) of 0.270 (95% CI: 0.115–0.424), which was statistically significant (t(10.10) = 3.88, *p* = 0.003). The 95% prediction interval was −0.166 to 0.705, indicating the expected range of true effects in future studies.

**Figure 3:**
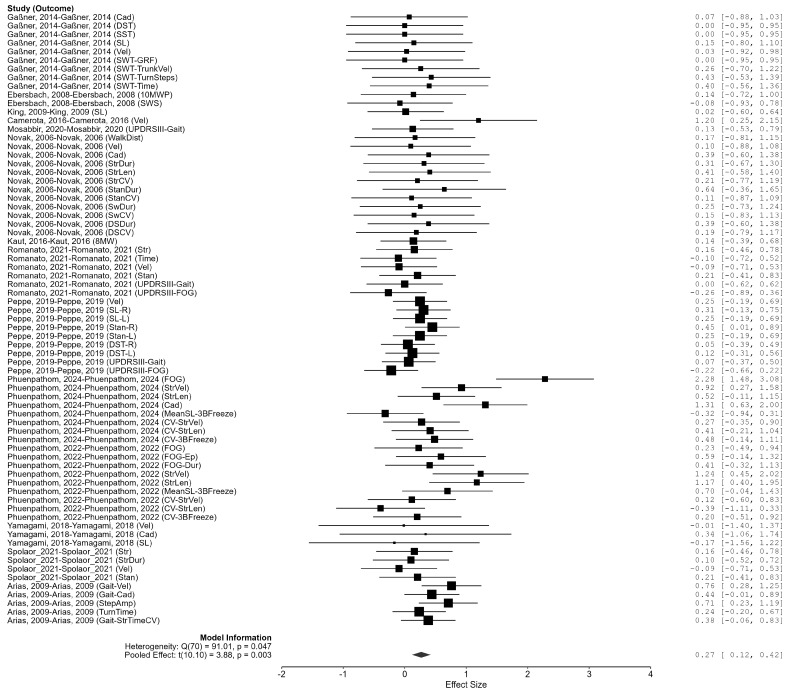
The Effect of Vibration Intervention on Gait in Parkinson’s Patients. Note. Data were derived from Gaßner et al. [87], Ebersbach et al. [86], King et al. [88], Camerota et al. [15], Mosabbir et al. [89], Novak et al. [16], Kaut et al. [78], Romanato et al. [92], Peppe et al. [59], Phuenpathom et al. [90,91], Yamagami et al. [94], Spolaor et al. [93], and Arias et al. [85].

Residual heterogeneity was significant (Q_e_(70) = 91.01, *p* = 0.047). In the multilevel variance decomposition, the between-study variance was estimated as τ^2^_study = 0.030 (τ_study = 0.175, 95% CI for τ: 0.063–0.345), and the within-study (effect-size-level) variance as τ^2^_within = 0.003 (τ_within = 0.055, 95% CI for τ: 0.000–0.242). Component-inclusion tests indicated that removing the study level worsened model fit (LRT = 6.096, *p* = 0.014), whereas removing the effect-size level did not materially change the fit (LRT = 0.023, *p* = 0.879), suggesting that most heterogeneity arises between studies, with within-study dependence being smaller but non-negligible (Figure 3).

**Figure 4:**
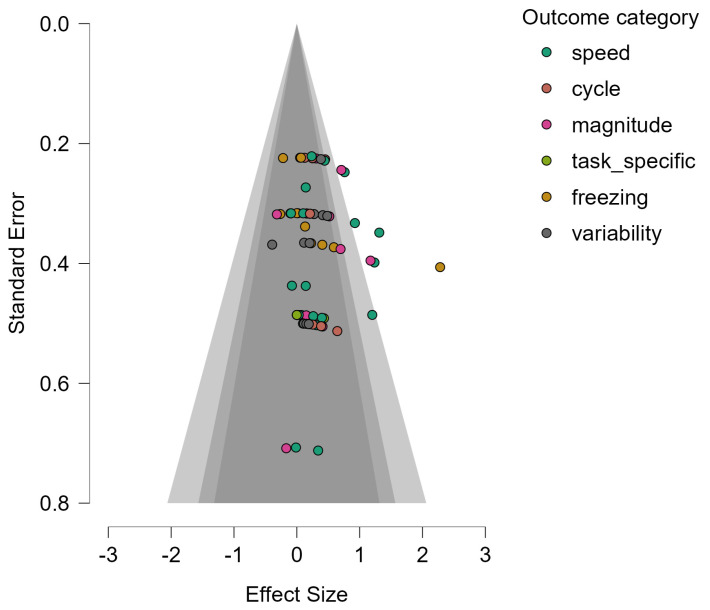
The funnel plot of included studies.

In the assessment of publication bias and small-study effects, two studies with missing data were excluded from the asymmetry analysis. The funnel plot (Figure 4) indicated that effect sizes were generally symmetrically distributed around the pooled estimate, and no significant evidence of asymmetry was detected in the funnel plot asymmetry test (Egger-type) (z = 0.582, *p* = 0.560), weighted regression (t(67) = 0.661, *p* = 0.511), or rank correlation test (Kendall’s τ = 0.154, *p* = 0.061). In addition, Rosenthal’s Fail-safe N was 1177, suggesting that a large number of unpublished studies would be required to negate the observed positive effect.

In the Trim-and-Fill analysis, no potentially missing studies were imputed (n = 0), and the adjusted pooled effect remained unchanged at g = 0.285 (95% CI: 0.194–0.376, *p* < 0.001). The between-study variance estimate also remained stable (τ = 0.183, *p* = 0.047), further supporting the minimal impact of publication bias on the overall effect estimate.

Finally, in the sensitivity analysis using the leave-one-out method, the pooled effect size estimate remained robust when individual studies were sequentially excluded, indicating that no single study disproportionately influenced the overall findings.

### 3.5. Meta-Regression Analysis of Clinical Moderators

**Figure 5:**
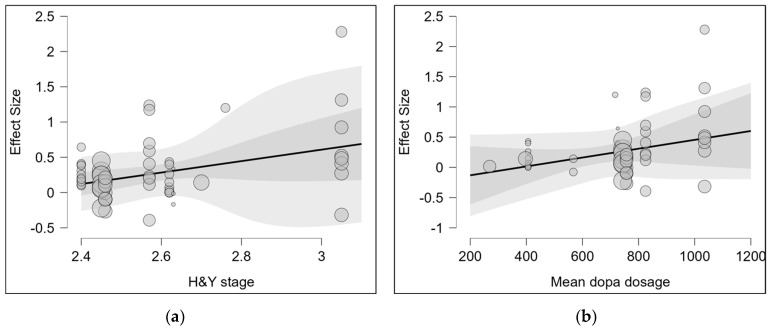
Bubble Plots for the Meta-Regression of (**a**) Hoehn and Yahr Stage and (**b**) Mean Levodopa Dosage on the Effect Size of Vibration Therapy for Gait. Note. Each dot represents the effect size of an individual study, with dot size proportional to the study’s weight in the meta-regression. The solid line indicates the fitted regression line, and the shaded area represents the 95% confidence interval.

A multilevel meta-regression was conducted to examine clinical moderators (Figure 5). First, in the analysis with the Hoehn and Yahr (H&Y) stage as a moderator, nine observations were excluded due to missing data, leaving 62 observations across 10 clusters (median = 4, max = 12). The pooled effect was significant (g = 0.266, 95% CI: 0.150–0.382, t(4.65) = 6.03, *p* = 0.002). The moderating effect of H&Y stage was also statistically significant (F_m_(1,1.66) = 45.32, *p* = 0.034). The regression coefficient was β(H&Y) = 0.811 (SE = 0.120, 95% CI: 0.176–1.446, t = 6.732), with an intercept of −1.825 (SE = 0.330, 95% CI: −3.349 to −0.300, *p* = 0.036). Residual heterogeneity was not significant (Q_e_(60) = 67.17, *p* = 0.245). These results indicate that higher H&Y stages were associated with significantly greater gait improvements following vibration intervention.

Next, in the analysis with mean levodopa dosage as a moderator, nine observations were again excluded, leaving 62 observations across 11 clusters (median = 3, max = 12). The pooled effect was significant (g = 0.252, 95% CI: 0.099–0.406, t(6.69) = 3.92, *p* = 0.006). However, the moderating effect of levodopa dosage did not reach statistical significance (F_m_(1,3.11) = 4.76, *p* = 0.114). The regression coefficient was β(mean dosage) = 7.335 × 10^−4^ (SE = 3.361 × 10^−4^, 95% CI: −3.155 × 10^−4^–0.002, *p* = 0.114), with an intercept of −0.278 (SE = 0.223, 95% CI: −0.962 to 0.405, *p* = 0.295). Residual heterogeneity was not significant (Q_e_(60) = 72.34, *p* = 0.132).

In summary, the multilevel model confirmed that clinical severity (H&Y stage) significantly moderated the gait effects of vibration intervention, whereas mean levodopa dosage did not show a significant moderating effect.

### 3.6. Subgroup Analysis of Intervention Moderators

A series of multilevel subgroup analyses was performed to further explore heterogeneity across study and intervention characteristics. Estimates are reported as pooled SMDs (Hedges’ g) with 95% confidence intervals (CI) and 95% prediction intervals (PI). Residual heterogeneity within each subgroup level was assessed using Q_e_ (df, *p*), and omnibus differences between levels were evaluated using Q_m_ (df, *p*) (Table 3).

**Figure 6:**
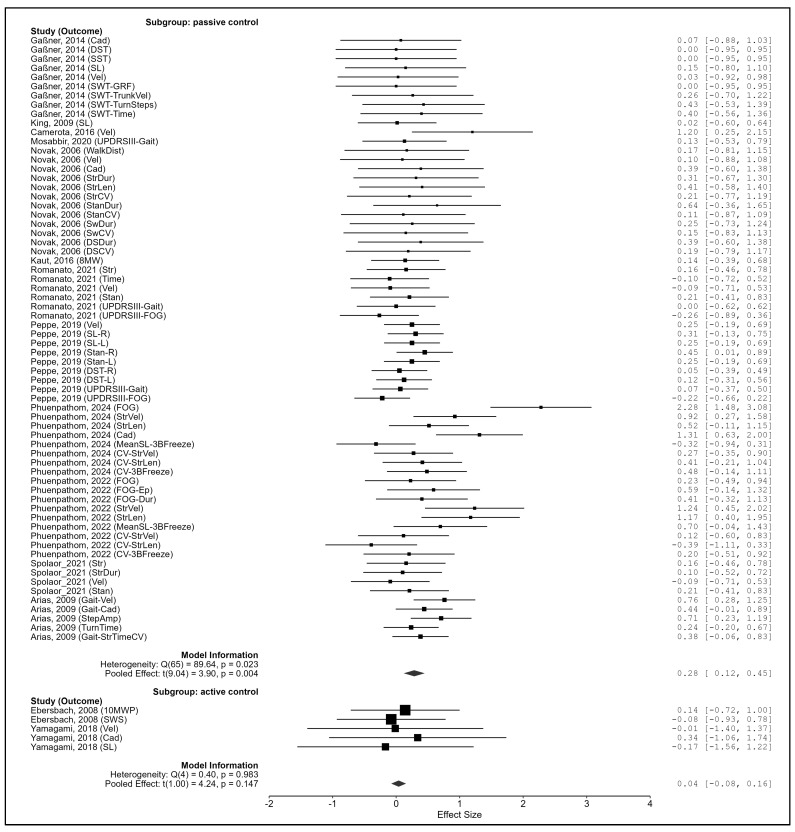
Subgroup analysis by control type. Note. Data were derived from Gaßner et al. [87], Ebersbach et al. [86], King et al. [88], Camerota et al. [15], Mosabbir et al. [89], Novak et al. [16], Kaut et al. [78], Romanato et al. [92], Peppe et al. [59], Phuenpathom et al. [90,91], Yamagami et al. [94], Spolaor et al. [93], and Arias et al. [85].

Control type. Interventions with passive control groups yielded significant pooled effects (g = 0.285, 95% CI: 0.120–0.450, *p* = 0.004; Q_e_(65) = 89.64, *p* = 0.023), whereas active controls did not (g = 0.039, 95% CI: −0.078–0.156, *p* = 0.147; Q_e_(4) = 0.40, *p* = 0.983). The between-group difference was statistically significant (Q_m_(1) = 11.13, *p* < 0.001) (Figure 6).

Amplitude type. No consistent subgroup effects were observed (≤1 mm: g = 0.351, *p* = 0.398; 1–5 mm: g = 0.147, *p* = 0.013; >5 mm: g = 0.068, *p* = 0.449). Differences between amplitude categories were not significant (Q_m_(2) = 2.51, *p* = 0.285).

**Figure 7:**
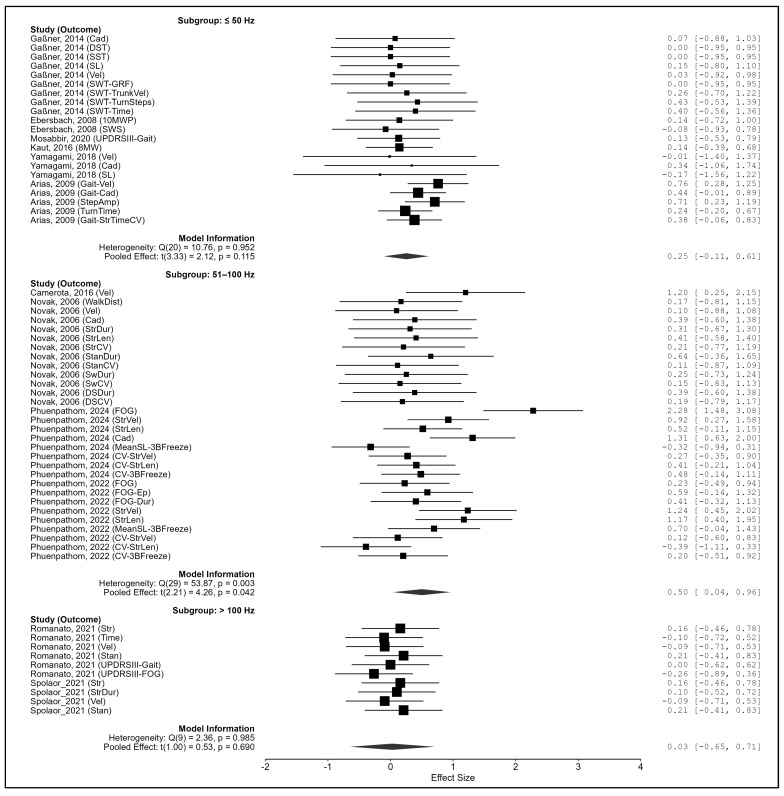
Subgroup analysis by frequency (Hz) type. Note. Data were derived from Gaßner et al. [87], Ebersbach et al. [86], King et al. [88], Camerota et al. [15], Mosabbir et al. [89], Novak et al. [16], Kaut et al. [78], Romanato et al. [92], Peppe et al. [59], Phuenpathom et al. [90,91], Yamagami et al. [94], Spolaor et al. [93], and Arias et al. [85].

Frequency type (Hz). Significant subgroup differences emerged (Q_m_(2) = 14.66, *p* < 0.001). The strongest effect was at 51–100 Hz (g = 0.502, 95% CI: 0.040–0.964, *p* = 0.042; Q_e_(29) = 53.87, *p* = 0.003), while ≤50 Hz (g = 0.253, *p* = 0.115) and >100 Hz (g = 0.028, *p* = 0.690) were not significant (Figure 7).

**Figure 8:**
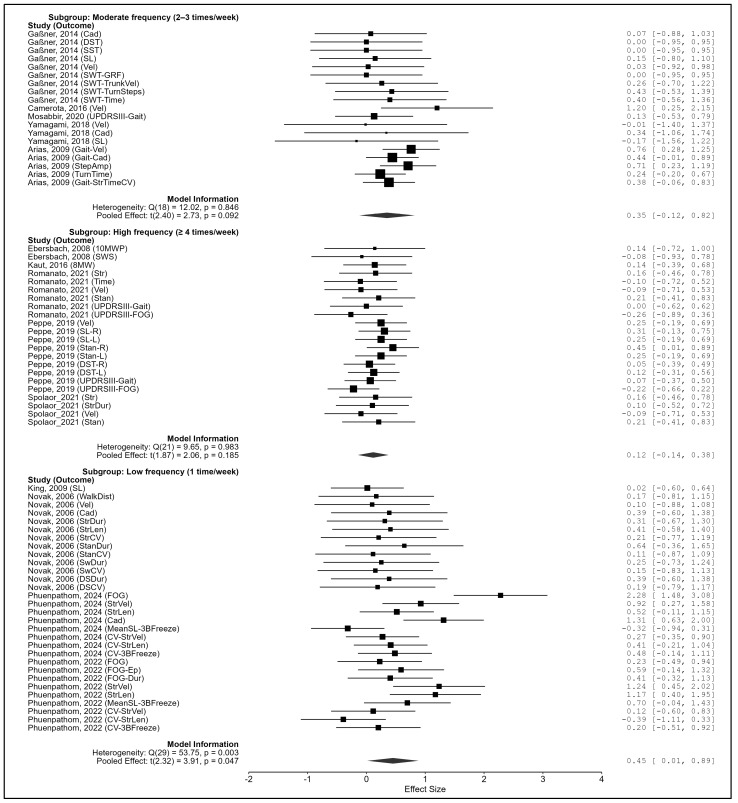
Subgroup analysis by weekly frequency type. Note. Data were derived from Gaßner et al. [87], Ebersbach et al. [86], King et al. [88], Camerota et al. [15], Mosabbir et al. [89], Novak et al. [16], Kaut et al. [78], Romanato et al. [92], Peppe et al. [59], Phuenpathom et al. [90,91], Yamagami et al. [94], Spolaor et al. [93], and Arias et al. [85].

Weekly frequency. Subgroup effects differed significantly (Q_m_(2) = 8.28, *p* = 0.016). Single weekly sessions produced significant improvement (g = 0.452, 95% CI: 0.015–0.888, *p* = 0.047), whereas moderate (2–3 times/week: g = 0.350, *p* = 0.092) and high frequencies (≥4 times/week: g = 0.117, *p* = 0.185) were not significant (Figure 8).

**Figure 9:**
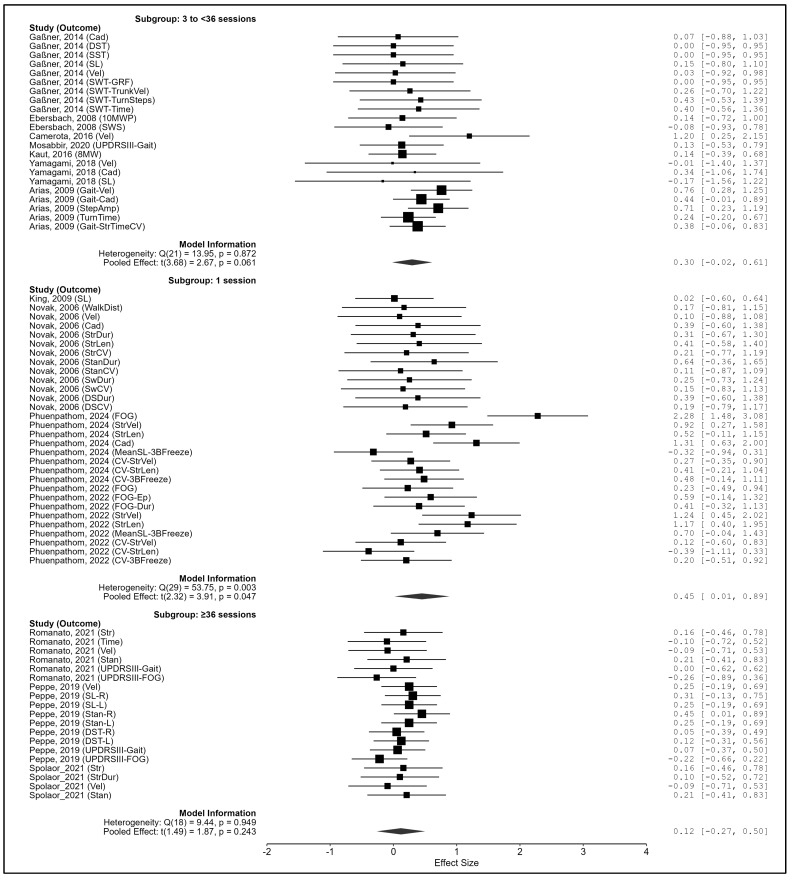
Subgroup analysis by total number of sessions. Note. Data were derived from Gaßner et al. [87], Ebersbach et al. [86], King et al. [88], Camerota et al. [15], Mosabbir et al. [89], Novak et al. [16], Kaut et al. [78], Romanato et al. [92], Peppe et al. [59], Phuenpathom et al. [90,91], Yamagami et al. [94], Spolaor et al. [93], and Arias et al. [85].

Total number of sessions. Subgroup differences were also significant (Q_m_(2) = 7.40, *p* = 0.025). Single-session protocols yielded significant effects (g = 0.457, 95% CI: 0.012–0.903, *p* = 0.047), 3–35 sessions showed a trend (g = 0.309, *p* = 0.074), and ≥36 sessions were non-significant (g = 0.119, *p* = 0.243) (Figure 9).

Intervention period. One-week interventions produced significant effects (g = 0.459, 95% CI: 0.109–0.808, *p* = 0.025), whereas longer protocols (2–7 weeks: g = 0.273, *p* = 0.192; ≥8 weeks: no convergence) did not. Subgroup differences were not significant (Q_m_(1) = 1.07, *p* = 0.302).

Total exposure time per session. None of the subgroup effects reached significance (≤3 min: g = 0.579, *p* = 0.130; 3–15 min: g = 0.293, *p* = 0.062; ≥15 min: g = 0.284, *p* = 0.388), and subgroup differences were not significant (Q_m_(2) = 3.53, *p* = 0.171).

Vibration type. Sinusoidal vibration produced significant pooled effects (g = 0.413, 95% CI: 0.119–0.706, *p* = 0.018; Q_e_(36) = 60.06, *p* = 0.007), while stochastic vibration did not (g = 0.187, *p* = 0.083). The between-group difference approached significance (Q_m_(1) = 2.86, *p* = 0.091).

Modality. Whole-body vibration (WBV) showed a non-significant effect (g = 0.287, *p* = 0.203), while focal mechanical vibration (FMV) was significant (g = 0.295, 95% CI: 0.055–0.536, *p* = 0.024). However, subgroup differences were not significant (Q_m_(1) = 0.00, *p* = 0.966).

Study design. Significant effects were found in RCTs (g = 0.316, 95% CI: 0.004–0.628, *p* = 0.048; Q_e_(36) = 70.57, *p* < 0.001), but not in randomized crossover (g = 0.149, *p* = 0.122) or non-RCTs (g = 0.384, *p* = 0.136). Differences across designs were marginal (Q_m_(2) = 5.48, *p* = 0.064).

**Figure 10:**
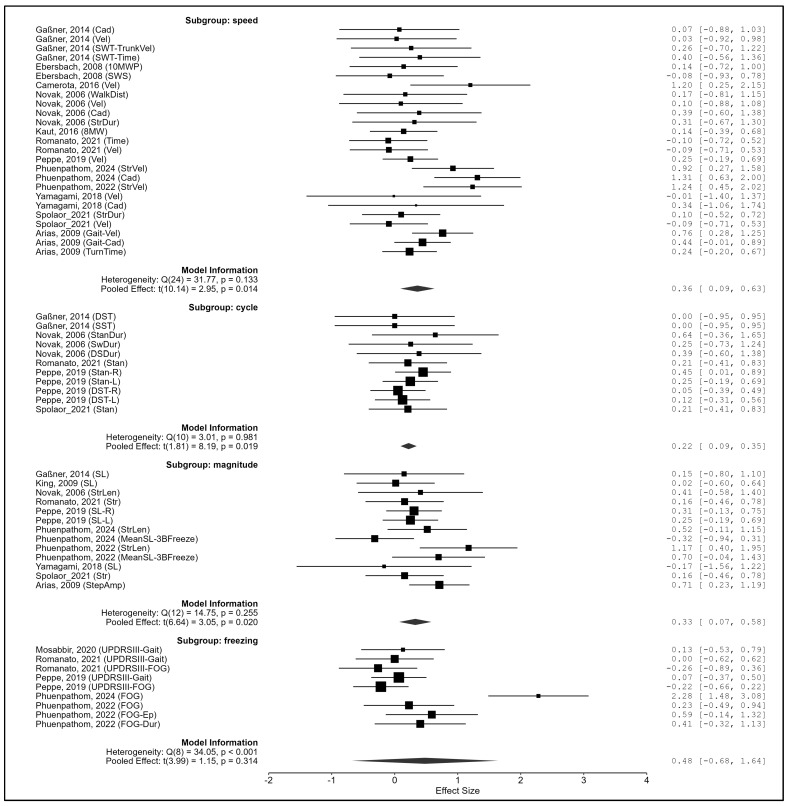
Subgroup analysis by outcome domain. Note. Data were derived from Gaßner et al. [87], Ebersbach et al. [86], King et al. [88], Camerota et al. [15], Mosabbir et al. [89], Novak et al. [16], Kaut et al. [78], Romanato et al. [92], Peppe et al. [59], Phuenpathom et al. [90,91], Yamagami et al. [94], Spolaor et al. [93], and Arias et al. [85].

Outcome domain. Subgroup differences were not significant overall (Q_m_(3) = 2.41, *p* = 0.491). However, significant pooled effects were observed for gait speed (g = 0.361, *p* = 0.014), cycle (g = 0.221, *p* = 0.019), and magnitude (g = 0.326, *p* = 0.020), whereas freezing of gait (g = 0.481, *p* = 0.314) and variability (no convergence) were not (Figure 10).

### 3.7. Sensitivity Analysis Restricted to Study Design

As a sensitivity analysis, we repeated the meta-analysis, including only randomized controlled trials (RCTs). The pooled effect size, estimated from 37 effect sizes across eight study clusters, was g = 0.316 (95% CI: 0.004–0.628, *p* = 0.048), which was statistically significant and consistent with the main analysis that included all studies. The 95% prediction interval ranged from −0.678 to 1.310, and between-study heterogeneity remained significant (Q_e_(36) = 70.57, *p* < 0.001). Variance decomposition revealed that the between-study variance (τ^2^ = 0.049, τ = 0.222) was smaller than, but comparable to, the within-study variance (τ^2^ = 0.090, τ = 0.301).

In the assessment of publication bias, the funnel plot appeared generally symmetrical. Egger’s regression (z = 0.688, *p* = 0.491), weighted regression (t(35) = 0.936, *p* = 0.356), and rank correlation (Kendall’s τ = 0.210, *p* = 0.069) were all non-significant, indicating no evidence of asymmetry. Rosenthal’s fail-safe N was 377, suggesting that a substantial number of unpublished null studies would be required to overturn the observed effect. Trim-and-fill analysis did not impute any missing studies (n = 0), and the adjusted effect estimate remained unchanged.

Subgroup analyses restricted to RCTs revealed several distinctive patterns. For vibration frequency, a significant effect was observed only in the ≤50 Hz category (g = 0.128, 95% CI: 0.035–0.221, *p* = 0.027), whereas effects in the 51–100 Hz (g = 0.607, *p* = 0.096) and >100 Hz categories were non-significant. The between-group difference was significant (Q_m_(1) = 15.60, *p* < 0.001), indicating that when considering only RCTs, relatively consistent improvements were observed under low-frequency stimulation. For weekly frequency, none of the individual subgroups reached significance (1 session/week: g = 0.580, *p* = 0.131; 2–3 sessions/week: g = 0.353, *p* = 0.310; ≥4 sessions/week: g = 0.016, *p* = 0.782), but the overall difference across categories was significant (Q_m_(2) = 20.01, *p* < 0.001). For the total number of sessions, the pooled effects of individual categories were either non-significant or borderline (3–35 sessions: g = 0.189, *p* = 0.096; 1 and ≥36 sessions: non-significant), yet subgroup differences were significant (Q_m_(1) = 7.82, *p* = 0.005).

Taken together, these results indicate that the overall positive effect of vibration interventions persisted even when restricting the analysis to RCTs, and further suggest that the magnitude of effects may vary according to specific intervention characteristics, particularly lower vibration frequency, the total number of sessions, and weekly frequency.

### 3.8. Certainty of Evidence for the Effects of Vibration Therapy on Gait in Patients with Parkinson’s Disease

The certainty of the evidence for the included studies was assessed using the GRADE approach (Table 4). The overall certainty of the evidence from the 11 randomized trials was rated as very low. The main reasons for downgrading were limitations in randomization procedures and allocation concealment, a serious risk of bias due to inadequate blinding of assessors, inconsistency arising from heterogeneous effect sizes across studies, and serious imprecision resulting from small sample sizes. In addition, indirectness was noted because many control groups employed active interventions such as exercise therapy rather than a placebo or no treatment, making it difficult to isolate the independent effects of vibration therapy. Thus, although RCT evidence suggests a potential benefit for gait function, the certainty of this evidence remains limited.

The certainty of the evidence from the three non-randomized trials was also rated as very low. All studies exhibited a serious risk of bias due to confounding factors and participant selection, and some reported extremely large effect sizes that represented severe inconsistencies compared with other studies. Moreover, the very small sample sizes further reduced confidence in the estimates.

In summary, while the available evidence suggests that vibration therapy may improve gait in patients with Parkinson’s disease, the overall certainty was rated as very low for both randomized and non-randomized studies. Therefore, current evidence remains limited and should be interpreted with caution.

## 4. Discussion

This meta-analysis synthesized evidence from 14 clinical trials, including randomized controlled and crossover designs, to evaluate the effects of vibration-based interventions on gait performance in individuals with Parkinson’s disease. The findings indicated that VT yielded small but statistically significant improvements in gait (Hedges’ g ≈ 0.285), with overall effect estimates consistently favoring intervention. Nevertheless, the modest magnitude of effect suggests that VT alone is unlikely to exert a substantial therapeutic impact, implying that while it may have utility in certain contexts, it should not be considered a comprehensive solution to gait impairment in Parkinson’s disease. Rather, its role may be more appropriately conceptualized as a supportive option within broader rehabilitation strategies.

Examination of specific outcome domains further clarified the scope of benefit. Significant improvements were observed in gait speed (g = 0.361), gait cycle (g = 0.221), and gait magnitude (g = 0.326). These effects are consistent with mechanistic hypotheses that vibration enhances proprioceptive input through stimulation of muscle spindles, thereby strengthening sensorimotor integration and facilitating rhythmicity, timing, and amplitude of locomotion [15,95]. By contrast, no significant changes were found in freezing of gait (FOG; g = 0.481, *p* = 0.314) or gait variability, suggesting that vibration therapy contributes primarily to basic gait functions while exerting limited influence on higher-order disturbances. Given that FOG is closely linked to impairments in executive function and motor planning, and gait variability is often associated with cognitive decline and instability of neural network coordination, it is unlikely that peripheral sensory stimulation alone can adequately address these domains [96,97,98]. This finding underscores the need to combine VT with cognitive training, neuromodulatory interventions, or other multimodal rehabilitation approaches when targeting more complex gait dysfunctions [90].

Subgroup analyses based on amplitude revealed no significant differences among the three categories (≤1 mm, 1–5 mm, >5 mm). This finding suggests that treatment efficacy is influenced more by vibration frequency and session structure than by amplitude itself. From a clinical standpoint, this has important implications, as improvements can be achieved even at low-to-moderate amplitudes, thereby enabling clinicians to prioritize safety when prescribing therapy. For vulnerable populations, such as older adults or individuals with osteoporosis who may be at increased risk from high-amplitude stimulation, the application of lower amplitudes represents a rational and practical strategy. Consistent with this perspective, low-magnitude, high-frequency vibration has been shown to support bone mineral density while ensuring safety [99]. Similarly, in a cohort of middle-aged and older women, variations in vibration posture and frequency combinations produced more pronounced modulation of lower limb muscle activity than amplitude alone [100]. Overall, these findings highlight the importance of parameters other than amplitude in determining the therapeutic efficacy of vibration interventions.

Synthesizing these results allows for the development of preliminary clinical recommendations. The observed benefits were modest in magnitude but most consistently present under conditions of intermediate frequency stimulation (51–100 Hz), brief exposure durations (≤3 min), and administration two to three times per week. Subgroup analyses further indicated that patients with higher Hoehn And Yahr stages showed significantly improvements, whereas the moderating effect of levodopa dosage did not reach statistical significance, leaving its influence on treatment response uncertain. Within these parameters, VT may serve as a supportive adjunct for enhancing fundamental gait performance. Nevertheless, its limited efficacy for freezing of gait and gait variability suggests that more complex impairments may require integrative approaches combining vibration with cognitive or neuromodulatory strategies.

Taken together, these findings suggest that VT can provide small but statistically significant improvements in fundamental aspects of gait in Parkinson’s disease, particularly in gait speed, cycle, and magnitude. The lack of amplitude-specific effects highlights the practicality of employing low-intensity protocols that balance safety and efficacy. Nonetheless, the absence of consistent benefits for freezing of gait and variability underscores the limitations of VT as a stand-alone treatment. Future research should focus on the development of standardized intervention protocols, large-scale randomized controlled trials, and the exploration of combined therapeutic approaches to optimize its role within the comprehensive management of Parkinson’s disease.

The study’s findings, while valuable, must be interpreted with caution due to several limitations. Primarily, the evidence base was constrained both in quantity and quality. The meta-analysis relied on a relatively small pool of studies, many of which were preliminary trials with limited statistical power, compromising the precision and external validity of the results. Critically, the reliability of the aggregated data is questionable, as a high risk of methodological bias was identified across numerous randomized controlled trials (RCTs), and all non-randomized studies carried a serious risk of bias. Furthermore, our comprehensive scope led to significant heterogeneity across the included interventions, encompassing diverse modalities (e.g., focal versus whole-body vibration) and study designs. Although statistical methods like multilevel meta-analysis were applied, this variability in protocols and outcome metrics remains a major factor contributing to the uncertainty. In particular, the heterogeneity of outcome measures ranging from basic gait parameters to functional mobility tests may have influenced the pooled estimates and contributed to variability in treatment effects. Additionally, the short-term nature of most interventions analyzed prevents definitive conclusions regarding the sustained, long-term efficacy of VT on gait function in Parkinson’s disease, highlighting the need for future trials with extended follow-up. Finally, although the pooled effect size (g ≈ 0.285) was statistically significant, it remains modest and does not in itself ensure clinical meaningfulness. Prior studies have suggested minimal clinically important differences (MCIDs) for specific gait outcomes in Parkinson’s disease, such as gait speed (≈0.05–0.22 m/s) [101], step length (≈3.6 cm) and gait variability (≈0.7%) [102], and Timed Up and Go (≈2–3 s) [103]. However, because we synthesized heterogeneous outcomes using SMDs, direct comparison with these raw-unit MCID thresholds was not feasible. This means that although our findings indicate small but significant improvements, they cannot be directly interpreted as exceeding clinically meaningful thresholds. Future studies focusing on single gait measures in raw units will be required to determine whether VT achieves changes that are clinically important in this population.

## 5. Conclusions

This meta-analysis provides evidence that VT yields modest yet statistically significant effects on fundamental gait parameters in patients with Parkinson’s disease. Specifically, pooled analyses demonstrated such improvements in gait speed, cadence, and stride length. In contrast, for functional outcomes such as FOG, no consistent or statistically significant effects were observed, indicating that the efficacy of VT for these complex domains remains uncertain. Subgroup and meta-regression analyses further indicated that treatment efficacy was influenced by clinical severity, medication status, and specific vibration parameters. In particular, protocols incorporating medium-frequency stimulation (51–100 Hz), brief session durations (≤3 min), and moderate administration frequency (two to three times per week) were associated with a modest yet significant effect. Taken together, these findings move beyond a simple binary statement of whether VT is effective, instead highlighting the differential effects of VT according to disease severity, medication status, and stimulation parameters.

Importantly, these results underscore the condition-dependent nature of VT efficacy and provide a solid foundation for the development of individualized VT protocols. By identifying both optimal stimulation parameters and the patient subgroups most likely to benefit, this study offers clinically actionable insights that emphasize the practical and translational implications of VT as a non-pharmacological and non-invasive adjunct to conventional rehabilitation. However, the clinical significance of these improvements has not yet been firmly established, and further research is required to determine their practical implications. Nevertheless, the observation that patients with greater disease severity demonstrated statistically significant effects suggests that VT may hold potential clinical value, particularly for individuals who are insufficiently responsive to standard therapies.

Nevertheless, the current body of evidence remains constrained by methodological heterogeneity, limited sample sizes, and variability in intervention protocols. Future research should therefore focus on large-scale, rigorously designed clinical trials to establish patient-specific stimulation parameters and to evaluate the long-term sustainability of therapeutic effects. Taken together, these findings provide an essential foundation for the integration of VT into evidence-based rehabilitation strategies for PD.

## Figures and Tables

**Table 1:** Outcome measures used in the included studies.

Category	Outcome Measures	Description
Gait speed	Cadence; Velocity; Stride velocity; Stride time; Step-walk-turn task—time to accomplish; Step-walk-turn task—trunk velocity; Time to walk 10 m; Stand-walk-sit time; Velocity over 24 h; 8-Meter Walk Test; Gait-Velocity; Gait-Cadence; Gait-Turn Time	Indicators of temporal performance reflecting how fast an individual walks, turns, or completes functional tasks.
Gait cycle	Double support time; Single support time; Stride duration; Stance duration; Swing duration; Stance Right/Left; Double support time Right/Left.	Measures of temporal phases within the gait cycle, quantifying stance, swing, and support periods.
Gait magnitude	Step length; Stride length; Stride length Right/Left; Stride; Gait-Step amplitude; Mean stride length of three strides before a freeze	Indicators of spatial amplitude of walking, including step and stride length, and stride size before freezing.
Gait variability	Stride coefficient of variation (CV); Stance CV; Swing CV; Double support CV; Stride velocity CV; Stride length CV; CV of three strides before a freeze; Gait-stride-time CV	Indices of consistency and stability of gait, calculated using the coefficient of variation in temporal or spatial parameters.
Freezing of gait (FOG)	Unified Parkinson’s Disease Rating Scale (UPDRS) Part III—gait item; UPDRS Part III—freezing of gait item; Freezing of gait occurrence; Freezing of gait episodes; Freezing duration; Mean stride length of three strides before a freeze; CV of three strides before a freeze	Clinical and objective measures capturing the occurrence, severity, and characteristics of freezing episodes.

**Table 2:** Study characteristics of 14 studies selected for the meta-analysis.

Author and Year	Study Type	Participants	Sample (N, Age)	Intervention and Control Protocol	Intervention Protocol	WBV Parameters	Posture and WBV Type	Device Type	Outcome Measures
Gaßner et al. (2014) [87]	RCT, SB	DD: 7.71 ± 5.28, H&Y: 2.62 ± 0.38, L-dopa: 406.35 ± 291.23	I: 8 (71.4 ± 4.4), C: 9 (68.2 ± 4.9)	I: independent WBV, C: Sham (Same platform and posture, vibration switched off)	Freq: 2–3/wk, Dur: 5 wk	Bouts: 5, Bout Dur: 1 min, Rest: 1 min, Freq: 6 Hz, Amp: 3 mm	Knees slightly bent	SRT Zeptor Medical plus noise	Cad; DST; SST; SL; Vel; SWT-GRF; SWT-TrunkVel; SWT-TurnSteps; SWT-Time
Ebersbach et al. (2008) [86]	RCT	DD: 7.26 ± 3.00, L-dopa: 567.62 ± 216.21	I: 10 (72.5 ± 6.0), C: 11 (75.0 ± 6.8)	I: WBV + conventional training, C: Active control (Conventional balance training)	Freq: 10/wk, Dur: 3 wk	Bouts: 1, Bout Dur: 15 min, Freq: 25 Hz, Amp: 7–14 mm	Slightly bent knees and hips	Galileo	10 MWP; SWS
King et al. (2009) [88]	RCO	DD: 6.80 ± 4.75, L-dopa: 269.39 ± 271.93	I: 20 (65.4 ± 9.9), C: 20 (65.4 ± 9.9)	I: independent WBV, C: Sham (Sat on Physioacoustic Chair without vibration)	Freq: 1/wk, Dur: 1 wk	Bouts: 5, Bout Dur: 1 min, Rest: 1 min	Seated in a chair	Physioacoustic Chair	SL
Camerota et al. (2016) [15]	RCT, SB	DD: 7.76 ± 4.64, H&Y: 2.76 ± 0.48, L-dopa: 716.47 ± 89.48	I: 10 (67.0 ± 7.96), C: 10 (65.5 ± 9.85)	I: independent FMV, C: Sham (Device placed near skin but not touching)	Freq: 3/wk, Dur: 1 wk	Bouts: 6, Bout Dur: 10 min, Rest: 1 min, Freq: 100 Hz, Amp: 0.2–0.5 mm	Supine and prone positions	Cro System; Nemoco SRL	Vel
Mosabbir et al. (2020) [89]	RCT, DB	DD: 6.50 ± 4.40	I: 21 (69.4 ± 9.5), C: 15 (69.4 ± 9.5)	I: independent FMV, C: Sham (Sat on identical chair, only 40 Hz humming sound)	Freq: 3/wk, Dur: 12 wk	Bouts: 6–8, Bout Dur: 2–3 min, Rest: 1 min, Freq: 40 Hz	Sitting posture	Physioacoustic reclining chair with 6 built-in speakers	UPDRSIII-Gait
Novak et al. (2006) [16]	WSC, NB	DD: 6.00 ± 3.90, H&Y: 2.40 ± 0.20, L-dopa: 725.70 ± 510.10	I: 8 (61.4 ± 12.4), C: 8 (58.9 ± 12.3)	I: independent FMV, C: sham (details not reported)	Freq: 1/wk, Dur: 1 wk	Bouts: 1, Bout Dur: 6 min, Freq: 70 Hz, Amp: 0.1–0.2 mm	Standing and walking posture	Wearable step-synchronized vibration device	WalkDist; Vel; Cad; StrDur; StrLen; StrCV; StanDur; StanCV; SwDur; SwCV; DSDur; DSCV
Kaut et al. (2016) [78]	RCT, DB	DD: 7.00 ± 5.85, H&Y: 2.70 ± 0.80, L-dopa: 396.88 ± 279.20	I: 29 (66.1 ± 8.28), C: 25 (67.9 ± 8.78)	I: independent WBV, C: sham (Same equipment/posture, lowest frequency)	Freq: 4/wk, Dur: 1 wk	Bouts: 6, Bout Dur: 1 min, Rest: 1 min, Freq: 7 Hz, Amp: 3 mm	Semi-squat posture	SR-Zeptor^®^	8 MW
Romanato et al. (2021) [92]	RCO, DB	DD: 11.88 ± 4.89, H&Y: 2.46 ± 0.51, L-dopa: 757.14 ± 290.88	I: 20 (67.46 ± 10.27), C: 20 (67.46 ± 10.27)	I: independent FMV, C: sham (Identical-appearing inactive device)	Freq: 5–6/wk, Dur: 8 wk	Bout Dur: 60–240 min, Freq: 300 Hz	Standing, walking, and static posture	Equistasi^®^	Str; Time; Vel; Stan; UPDRSIII-Gait; UPDRSIII-FOG
Peppe et al. (2019) [59]	RCO, DB	DD: 8.35 ± 3.60, H&Y: 2.45 ± 0.50, L-dopa: 743.30 ± 293.00	I: 40 (61.36 ± 9.9), C: 40 (61.36 ± 9.9)	I: independent FMV, C: sham (Identical device without vibration)	Freq: 5–6/wk, Dur: 8 wk	Bout Dur: 240 min	-	Equistasi^®^	Vel; SL-R; SL-L; Stan-R; Stan-L; DST-R; DST-L; UPDRSIII-Gait; UPDRSIII-FOG
Phuenpathom et al. (2024) [90]	RCT, SB	DD: 12.25 ± 3.16, H&Y: 3.05 ± 0.59, L-dopa: 1035.30 ± 350.96	I: 20 (72.0 ± 7.2), C: 20 (71.2 ± 7.2)	I: independent FMV, C: sham (Identical insoles with battery removed)	Freq: 1/wk, Dur: 1 wk	Bouts: 1, Bout Dur: 1.6 min, Freq: 100 Hz	Seated	FOG shoes	FOG; StrVel; StrLen; Cad; MeanSL-3BFreeze; CV-StrVel; CV-StrLen; CV-3BFreeze
Phuenpathom et al. (2022) [91]	RCT	DD: 10.26 ± 2.79, H&Y: 2.57 ± 0.47, L-dopa: 825.01 ± 372.27	I: 15 (63.26 ± 4.78), C: 15 (60.4 ± 5.87)	I: independent FMV, C: no stimulation	Freq: 1/wk, Dur: 1 wk	Bouts: 1, Bout Dur: 1.6 min, Freq: 100 Hz	-	Custom-made vibratory eccentric mass device	FOG; FOG-Ep; FOG-Dur; StrVel; StrLen; MeanSL-3BFreeze; CV-StrVel; CV-StrLen; CV-3BFreeze
Yamagami et al. (2018) [94]	2G, Pilot	DD: 5.63 ± 5.15, H&Y: 2.63 ± 1.06	I: 4 (69.75 ± 4.57), C: 4 (65.25 ± 8.18)	I: WBV combined with agility training, C: agility training	Freq: 3/wk, Dur: 5 wk	Bouts: 3, Bout Dur: 1–3 min, Freq: 18 Hz, Amp: 5.2 mm	Standing in a semi-squat posture	Galileo Med L	Vel; Cad; SL
Spolaor et al. (2021) [93]	RCO, DB	DD: 11.88 ± 3.23, H&Y: 2.46 ± 0.51, L-dopa: 757.14 ± 290.88	I: 20 (67.46 ± 10.27), C: 20 (67.46 ± 10.27)	I: independent FMV, C: sham (Identical kit not delivering vibration)	Freq: 5–6/wk, Dur: 8 wk	Bout Dur: 60–240 min, Freq: 9000 Hz	-	Equistasi^®^	Str; StrTime; Vel; Stan
Arias et al. (2009) [85]	DB	-	I: 10 (66.9 ± 11.11), C: 11 (66.55 ± 5.57)	I: independent WBV, C: sham (Platform identical, vibration switched off)	Freq: 2–3/wk, Dur: 5 wk	Bouts: 5, Bout Dur: 1 min, Rest: 1 min, Freq: 6 Hz	Standing with slightly bent knees and feet firmly placed	Fit Massage	Vel; Cad; StepAmp; TurnTime; StrTimeCV

Abbreviations: I, Intervention; C, Control; DD, Disease duration (years); H&Y, Hoehn and Yahr stage; L-dopa, Mean levodopa dosage (mg/day); RCT, Randomized controlled trial; SB, Single-blind; DB, Double-blind; RCO, Randomized crossover trial; WSC, Within-subject comparison; NB, Not blinded; 2G, Two-group; Pilot, Pilot study; WBV, Whole-body vibration; FMV, Focal muscle vibration; Freq, Frequency; Dur, Duration; wk, Week; Bout Dur, Bout duration; Rest, Rest period between bouts; Amp, Amplitude; Cad, Cadence; DST, Double support time; SST, Single support time; SL, Step length; Vel, Velocity; SWT, Step-walk-turn task; SWT-GRF, Resulting ground reaction force in stepping down; SWT-TrunkVel, Resulting velocity of the trunk in stepping down; SWT-TurnSteps, Steps for turning in SWT task; SWT-Time, Time to accomplish the SWT task; 10 MWP, 10 m walking performance; SWS, Stand-walk-sit; WalkDist, Walking distance; StrDur, Stride duration; StrLen, Stride length; StrCV, Stride coefficient of variation; StanDur, Stance duration; StanCV, Stance coefficient of variation; SwDur, Swing duration; SwCV, Swing coefficient of variation; DSDur, Double support duration; DSCV, Double support coefficient of variation; Str, Stride; Stan, Stance; UPDRSIII, Unified Parkinson’s Disease Rating Scale part III; UPDRSIII-Gait, UPDRSIII gait score; UPDRSIII-FOG, UPDRSIII freezing of gait score; FOG, Freezing of gait; FOG-Ep, Freezing episodes; FOG-Dur, Freezing duration; StrVel, Stride velocity; MeanSL-3BFreeze, Mean stride lengths of three strides before a freeze; CV-StrVel, Coefficient of variation in stride velocity; CV-StrLen, Coefficient of variation in stride length; CV-3BFreeze, Coefficient of variation in three strides before a freeze; SL-R, Step length right; SL-L, Step length left; Stan-R, Stance right; Stan-L, Stance left; DST-R, Double support time right; DST-L, Double support time left; StepAmp, Step amplitude; TurnTime, Turn time; StrTimeCV, Stride-time coefficient of variation.

**Table 3:** Results of Multilevel Subgroup Analyses for Moderator Variables.

Moderator Variable	Subgroup Level	g (Hedges’ g)	95% CI	95% PI	Q_e_(df), *p*
Control type	Passive control	0.285 **	[0.120, 0.450]	[−0.185, 0.755]	Q_m_(1) = 11.13, <0.001 **
	Active control	0.039	[−0.078, 0.156]	[−0.078, 0.156]	
Amplitude	≤1 mm	0.351	[−2.864, 3.566]	[−2.864, 3.566]	Q_m_(2) = 2.51, 0.285
	1–5 mm	0.147 *	[0.109, 0.184]	[0.109, 0.184]	
	>5 mm	0.068	[−0.666, 0.801]	[−0.666, 0.801]	
Frequency (Hz)	≤50 Hz	0.253	[−0.106, 0.613]	[−0.319, 0.826]	Q_m_(2) = 14.66, <0.001 **
	51–100 Hz	0.502 *	[0.040, 0.964]	[−1.085, 2.089]	
	>100 Hz	0.028	[−0.651, 0.708]	[−0.651, 0.708]	
Weekly frequency	1 time/week	0.452 *	[0.015, 0.888]	[−1.049, 1.952]	Q_m_(2) = 8.28, 0.016 *
	2–3 times/week	0.35	[−0.123, 0.823]	[−0.423, 1.124]	
	≥4 times/week	0.117	[−0.145, 0.378]	[−0.145, 0.378]	
Total sessions	1 session	0.457 *	[0.012, 0.903]	[−1.006, 1.920]	Q_m_(2) = 7.40, 0.025 *
	3–35 sessions	0.309	[−0.053, 0.671]	[−0.183, 0.801]	
	≥36 sessions	0.119	[−0.266, 0.503]	[−0.266, 0.503]	
Intervention period	1 week	0.459 *	[0.109, 0.808]	[−0.808, 1.726]	Q_m_(1) = 1.07, 0.302
	2–7 weeks	0.273	[−0.314, 0.859]	[−0.648, 1.193]	
	≥8 weeks	- (no convergence)	-	-	
Total exposure/session	≤3 min	0.579	[−0.946, 2.104]	[−5.809, 6.966]	Q_m_(2) = 3.53, 0.171
	3–15 min	0.293	[−0.026, 0.613]	[−0.123, 0.710]	
	≥15 min	0.284	[−0.957, 1.525]	[−0.994, 1.562]	
Vibration type	Sinusoidal	0.413 *	[0.119, 0.706]	[−0.637, 1.462]	Q_m_(1) = 2.86, 0.091
	Stochastic	0.187	[−0.037, 0.412]	[−0.267, 0.642]	
Modality	WBV	0.287	[−0.321, 0.895]	[−0.478, 1.053]	Q_m_(1) = 0.00, 0.966
	FMV	0.295 *	[0.055, 0.536]	[−0.343, 0.933]	
Study design	RCT	0.316 *	[0.004, 0.628]	[−0.678, 1.310]	Q_m_(2) = 5.48, 0.064
	RCO	0.149	[−0.178, 0.476]	[−0.178, 0.476]	
	nRCT	0.384	[−0.493, 1.261]	[−0.736, 1.504]	
Outcome domain	Speed	0.361 *	[0.089, 0.633]	[−0.405, 1.127]	Q_m_(3) = 2.41, 0.491
	Cycle	0.221 *	[0.092, 0.349]	[0.092, 0.349]	
	Magnitude	0.326 *	[0.071, 0.582]	[−0.141, 0.794]	
	Freezing of gait	0.481	[−0.682, 1.644]	[−2.280, 3.242]	
	Variability	- (no convergence)	-	-	

Note. Estimates are from multilevel random-effects models (REML) with effect sizes nested within studies. For each subgroup, pooled effect sizes are expressed as Hedges’ g with 95% CIs and 95% PIs, along with residual heterogeneity (Q_e_, df, *p*). Differences across subgroup levels were tested using omnibus Q_m_ statistics (df, *p*). - * *p* < 0.05; ** *p* < 0.01. Total number of sessions = frequency × duration (weeks). Total exposure time per session = bout duration (minutes) × number of bouts per session.

**Table 4:** GRADE assessment of the certainty of evidence for vibration therapy on gait outcomes in Parkinson’s disease.

Certainty Assessment							№ of Patients		Effect		Certainty	Importance
№ of Studies	Study Design	Risk of Bias	Inconsistency	Indirectness	Imprecision	Other Considerations	Vibration Therapy	Sham, Active Control, or Usual Care	Relative (95% CI)	Absolute (95% CI)		
RCT and RCO												
11	randomized trials	very serious	serious ^a^	not serious	serious ^b^	none	-/213	-/214	not estimable		⨁◯◯◯ Very low ^a,b^	IMPORTANT
Non RCT												
3	non-randomized studies	extremely serious ^c^	not serious	not serious	serious ^d^	none	-/22	-/22	not estimable		⨁◯◯◯ Very low ^c,d^	IMPORTANT

Explanations: ^a.^ Considerable statistical heterogeneity was observed (Q_e_(50) = 81.13, *p* < 0.01). Although effect estimates were generally positive in direction, the magnitude varied and some subgroup effects differed, indicating inconsistency not fully explained by moderators. ^b.^ The total sample size was only 11 participants, which is far below the optimal information size. The resulting confidence interval was wide and included both clinically meaningful benefit and the possibility of no effect, indicating serious imprecision. ^c.^ All three non-randomized studies were rated as high risk of bias using ROBINS-I, mainly due to confounding and selection bias. Although several RCTs had low or some concerns of bias, the inclusion of multiple high-risk non-RCTs lowered our confidence in the overall evidence, and we therefore downgraded by one level for risk of bias. ^d.^ The total sample size was only 22 participants, which is far below the optimal information size. The resulting confidence interval was wide and included both clinically meaningful benefit and the possibility of no effect, indicating serious imprecision.

## Data Availability

The data supporting the findings of this study are available from the corresponding author upon reasonable request.

## Supplementary Materials

The following supporting information can be downloaded at: https://www.mdpi.com/article/10.3390/biomedicines13102505/s1, File S1: The PRISMA 2020 checklist.

## References

[1] Müller T., Möhr J.-D. (2018). Long-term management of Parkinson’s disease using levodopa combinations. Expert Opin. Pharmacother..

[2] Schütz L., Sixel-Döring F., Hermann W. (2022). Management of sleep disturbances in Parkinson’s disease. J. Park. Dis..

[3] Zhu J., Cui Y., Zhang J., Yan R., Su D., Zhao D., Wang A., Feng T. (2024). Temporal trends in the prevalence of Parkinson’s disease from 1980 to 2023: A systematic review and meta-analysis. Lancet Healthy Longev..

[4] Chaudhuri K.R., Azulay J.-P., Odin P., Lindvall S., Domingos J., Alobaidi A., Kandukuri P.L., Chaudhari V.S., Parra J.C., Yamazaki T. (2024). Economic burden of Parkinson’s disease: A multinational, real-world, cost-of-illness study. Drugs-Real World Outcomes.

[5] Moustafa A.A., Chakravarthy S., Phillips J.R., Gupta A., Keri S., Polner B., Frank M.J., Jahanshahi M. (2016). Motor symptoms in Parkinson’s disease: A unified framework. Neurosci. Biobehav. Rev..

[6] Thanvi B., Lo N., Robinson T. (2007). Levodopa-induced dyskinesia in Parkinson’s disease: Clinical features, pathogenesis, prevention and treatment. Postgrad. Med. J..

[7] Mueller T., Russ H. (2006). Levodopa, motor fluctuations and dyskinesia in Parkinson’s disease. Expert Opin. Pharmacother..

[8] Pickering R.M., Fitton C., Ballinger C., Fazakarley L., Ashburn A. (2013). Self reported adherence to a home-based exercise programme among people with Parkinson’s disease. Park. Relat. Disord..

[9] Ha J., Park J.H., Lee J.S., Kim H.Y., Song J.O., Yoo J., Ahn J.H., Youn J., Cho J.W. (2024). Effectiveness of Live-Streaming Tele-Exercise Intervention in Patients With Parkinson’s Disease: A Pilot Study. J. Mov. Disord..

[10] Li J., Aulakh N., Culum I., Roberts A.C. (2024). Adherence to Non-Pharmacological Interventions in Parkinson’s Disease: A Rapid Evidence Assessment of the Literature. J. Park. Dis..

[11] Goodwin G.M., McCloskey D.I., Matthews P.B. (1972). Proprioceptive illusions induced by muscle vibration: Contribution by muscle spindles to perception?. Science.

[12] Lapole T., Tindel J. (2015). Acute effects of muscle vibration on sensorimotor integration. Neurosci. Lett..

[13] Forner-Cordero A., Steyvers M., Levin O., Alaerts K., Swinnen S.P. (2008). Changes in corticomotor excitability following prolonged muscle tendon vibration. Behav. Brain Res..

[14] Ritzmann R., Gollhofer A., Kramer A. (2013). The influence of vibration type, frequency, body position and additional load on the neuromuscular activity during whole body vibration. Eur. J. Appl. Physiol..

[15] Camerota F., Celletti C., Suppa A., Galli M., Cimolin V., Filippi G.M., La Torre G., Albertini G., Stocchi F., De Pandis M.F. (2016). Focal muscle vibration improves gait in Parkinson’s disease: A pilot randomized, controlled trial. Mov. Disord. Clin. Pract..

[16] Novak P., Novak V. (2006). Effect of step-synchronized vibration stimulation of soles on gait in Parkinson’s disease: A pilot study. J. Neuroeng. Rehabil..

[17] Dincher A., Schwarz M., Wydra G. (2019). Analysis of the Effects of Whole—Body Vibration in Parkinson Disease–Systematic Review and Meta—Analysis. Pm&r.

[18] Fischer M., Vialleron T., Laffaye G., Fourcade P., Hussein T., Chèze L., Deleu P.-A., Honeine J.-L., Yiou E., Delafontaine A. (2019). Long-term effects of whole-body vibration on human gait: A systematic review and meta-analysis. Front. Neurol..

[19] Arenales Arauz Y.L., Ahuja G., Kamsma Y.P., Kortholt A., van der Zee E.A., van Heuvelen M.J. (2022). Potential of whole-body vibration in Parkinson’s disease: A systematic review and meta-analysis of human and animal studies. Biology.

[20] Zhao Y.-G., Lv W., Huo H.-Q., Wu J.-R., Cheng W.-W., Wang S. (2023). Meta-analysis of the effect of whole-body vibration training on the improvement of limb function in patients with Parkinson’s disease. Eur. Rev. Med. Pharmacol. Sci..

[21] Page M.J., McKenzie J.E., Bossuyt P.M., Boutron I., Hoffmann T.C., Mulrow C.D., Shamseer L., Tetzlaff J.M., Akl E.A., Brennan S.E. (2021). The PRISMA 2020 statement: An updated guideline for reporting systematic reviews. bmj.

[22] Higgins J.P., Altman D.G., Gøtzsche P.C., Jüni P., Moher D., Oxman A.D., Savović J., Schulz K.F., Weeks L., Sterne J.A. (2011). The Cochrane Collaboration’s tool for assessing risk of bias in randomised trials. bmj.

[23] Chandler J., Cumpston M., Li T., Page M.J., Welch V. (2019). Cochrane Handbook for Systematic Reviews of Interventions.

[24] Oroszi T., Van Heuvelen M.J., Nyakas C., Van Der Zee E.A. (2020). Vibration detection: Its function and recent advances in medical applications. F1000Research.

[25] Moore T.H., Higgins J.P., Dwan K. (2023). Ten tips for successful assessment of risk of bias in randomized trials using the RoB 2 tool: Early lessons from Cochrane. Cochrane Evid. Synth. Methods.

[26] Higgins J.P., Savović J., Page M.J., Elbers R.G., Sterne J.A., Higgins J., Thomas J., Chandler J., Cumpston M., Li T., Page M., Welch V. (2019). Assessing risk of bias in a randomized trial. Cochrane Handbook for Systematic Reviews of Interventions.

[27] Morris S.B. (2008). Estimating effect sizes from pretest-posttest-control group designs. Organ. Res. Methods.

[28] Morris S.B., DeShon R.P. (2002). Combining effect size estimates in meta-analysis with repeated measures and independent-groups designs. Psychol. Methods.

[29] Fu R., Vandermeer B.W., Shamliyan T.A., O’Neil M.E., Yazdi F., Fox S.H., Morton S.C. (2013). Handling Continuous Outcomes in Quantitative Synthesis.

[30] Hedges L.V. (1981). Distribution theory for Glass’s estimator of effect size and related estimators. J. Educ. Stat..

[31] Cooper H., Hedges L.V., Valentine J.C. (2019). The Handbook of Research Synthesis and Meta-Analysis.

[32] Hox J.J., De Leeuw E.D. (2003). Multilevel models for meta-analysis. Multilevel Modeling.

[33] Del Re A. (2015). A practical tutorial on conducting meta-analysis in R. Quant. Methods Psychol..

[34] Fernández-Castilla B., Declercq L., Jamshidi L., Beretvas S.N., Onghena P., Van den Noortgate W. (2021). Detecting selection bias in meta-analyses with multiple outcomes: A simulation study. J. Exp. Educ..

[35] Simental-Mendia L.E., Cicero A.F., Atkin S.L., Majeed M., Sahebkar A. (2019). A systematic review and meta-analysis of the effect of curcuminoids on adiponectin levels. Obes. Res. Clin. Pract..

[36] Wang N. (2023). Conducting meta-analyses of proportions in R. J. Behav. Data Sci..

[37] Orwin R.G. (1983). A fail-safe N for effect size in meta-analysis. J. Educ. Stat..

[38] Duval S., Tweedie R. (2000). A nonparametric “trim and fill” method of accounting for publication bias in meta-analysis. J. Am. Stat. Assoc..

[39] Lendraitienė E., Rėkus E., Volkevičiūtė A., Tunaitytė A., Venslauskas M., Abramavičius S., Stankevičius E. (2024). Research of upper limb tremor reduction with a vibrational medical device for parkinson’s disease. Technol. Disabil..

[40] Jöbges E., Elek J., Rollnik J., Dengler R., Wolf W. (2002). Vibratory proprioceptive stimulation affects Parkinsonian tremor. Park. Relat. Disord..

[41] Ronconi G., Gatto D.M., Ariani M., Codazza S., Panunzio M., Coraci D., Ferrara P.E. (2024). Effects of focal muscle vibration on cervical pain in Parkinson’s disease patients: A pilot study. Eur. J. Transl. Myol..

[42] Burke D., Andrews C.J., Lance J.W. (1972). Tonic vibration reflex in spasticity, Parkinson’s disease, and normal subjects. J. Neurol. Neurosurg. Psychiatry.

[43] Aggarwal R., Pretzer-Aboff I., Winfree K.N., Agrawal S.K., Behari M. (2019). Clinical outcomes of step-synchronized vibration training in patients of Parkinson’s disease with freezing of gait. Ann. Mov. Disord..

[44] Valkovič P., Krafczyk S., Bötzel K. (2006). Postural reactions to soleus muscle vibration in Parkinson’s disease: Scaling deteriorates as disease progresses. Neurosci. Lett..

[45] Ghoseiri K., Forogh B., Ali Sanjari M., Bavi A. (2009). Effects of vibratory orthosis on balance in idiopathic Parkinson’s disease. Disabil. Rehabil. Assist. Technol..

[46] Goetz C.G. (2009). Jean-Martin Charcot and his vibratory chair for Parkinson disease. Neurology.

[47] Harris M.A., Marion S.A., Spinelli J.J., Tsui J.K., Teschke K. (2012). Occupational exposure to whole-body vibration and parkinson’s disease: Results from a population-based case-control study. Am. J. Epidemiol..

[48] Valkovič P., Krafczyk S., Šaling M., Benetin J., Bötzel K. (2006). Postural reactions to neck vibration in Parkinson’s disease. Mov. Disord..

[49] Kammermeier S., Dietrich L., Maierbeck K., Plate A., Lorenzl S., Singh A., Bötzel K. (2017). Neck vibration proprioceptive postural response intact in progressive supranuclear palsy unlike idiopathic Parkinson’s disease. Front. Neurol..

[50] Silveira-Ciola A.P., Barbieri F.A., Soares C.F., Marques N.R., Simieli L., Faganello-Navega F.R. (2023). The effect of whole body vibration on gait stability in individuals with Parkinson’s disease: A preliminary study. Int. J. Ther. Rehabil..

[51] Rickards C., Cody F. (1997). Proprioceptive control of wrist movements in Parkinson’s disease. Reduced muscle vibration-induced errors. Brain.

[52] Boddy A., Barta K., Flores M., Sawyer K., Perry L., Campbell A. (2025). Immediate impact of whole-body vibration on backward walking in individuals with Parkinson disease. Physiother. Theory Pract..

[53] Winfree K.N., Pretzer-Aboff I., Hilgart D., Aggarwal R., Behari M., Agrawal S.K. (2013). The effect of step-synchronized vibration on patients with Parkinson’s disease: Case studies on subjects with freezing of gait or an implanted deep brain stimulator. IEEE Trans. Neural Syst. Rehabil. Eng..

[54] Han J., Jung J., Lee J., Kim E., Lee M., Lee K. (2013). Effect of muscle vibration on postural balance of Parkinson’s diseases patients in bipedal quiet standing. J. Phys. Ther. Sci..

[55] Varalta V., Righetti A., Evangelista E., Vantini A., Martoni A., Tamburin S., Fonte C., Di Vico I.A., Tinazzi M., Waldner A. (2024). Effects of upper limb vibratory stimulation training on motor symptoms in Parkinson’s disease: An observational study. J. Rehabil. Med..

[56] Chouza M., Arias P., Viñas S., Cudeiro J. (2011). Acute effects of whole—Body vibration at 3, 6, and 9 hz on balance and gait in patients with Parkinson’s disease. Mov. Disord..

[57] Chang C.-M., Tsai C.-H., Lu M.-K., Tseng H.-C., Lu G., Liu B.-L., Lin H.-C. (2022). The neuromuscular responses in patients with Parkinson’s disease under different conditions during whole-body vibration training. BMC Complement. Med. Ther..

[58] Garção D.C., dos Santos M.R.H., da Silva Correia A.G., Cajueiro C.A.G., de Oliveira J.S., Fraga B.P., Moreira O.S.M. (2022). Influence of whole-body vibration and gait training with additional load on functioning, balance, and gait in patients with Parkinson’s disease. Res. Soc. Dev..

[59] Peppe A., Paone P., Paravati S., Baldassarre M., Bakdounes L., Spolaor F., Guidotto A., Pavan D., Sawacha Z., Clerici D. (2019). Proprioceptive focal stimulation (Equistasi^®^) may improve motor symptoms in moderate Parkinson’s disease patients Italian multicentric preliminary open study. AGE.

[60] Cen S., Ma J., Sun H., Zhang H., Li Y., Mao W., Xu E., Mei S., Chhetri J.K., Ruan Z. (2024). Vibrotactile Foot Device for Freezing of Gait in Parkinson’s Disease: A Pilot Study. Mov. Disord. Clin. Pract..

[61] Ferrara P.E., Gatto D.M., Codazza S., Zordan P., Stefinlongo G., Coraci D., Lo Monaco M.R., Ricciardi D., Ronconi G. (2022). Effects of focal muscle vibration on gait and balance in Parkinson patients: Preliminary results. Appl. Sci..

[62] Rossi S., Lisini Baldi T., Aggravi M., Ulivelli M., Cioncoloni D., Niccolini V., Donati L., Prattichizzo D. (2020). Wearable haptic anklets for gait and freezing improvement in Parkinson’s disease: A proof-of-concept study. Neurol. Sci..

[63] Haas C.T., Turbanski S., Kessler K., Schmidtbleicher D. (2006). The effects of random whole-body-vibration on motor symptoms in Parkinson’s disease. NeuroRehabilitation.

[64] Soares L.T., Pereira A.J.F., Magno L.D.P., Figueiras H.d.M., Sobral L.L. (2014). Balance, gait and quality of life in Parkinson’s disease: Effects of whole body vibration treatment. Fisioter. Mov..

[65] Larocque K.A. (2015). The Effect of Acute Muscle Tendon Vibration on Motor Unit Activity in the Contralateral, More-Affected Limb in Parkinson’s Disease. Master’s Thesis.

[66] Pereira M.P., Gobbi L.T., Almeida Q.J. (2016). Freezing of gait in Parkinson’s disease: Evidence of sensory rather than attentional mechanisms through muscle vibration. Park. Relat. Disord..

[67] De Nunzio A.M., Nardone A., Picco D., Nilsson J., Schieppati M. (2008). Alternate trains of postural muscle vibration promote cyclic body displacement in standing parkinsonian patients. Mov. Disord..

[68] Volpe D., Giantin M.G., Fasano A. (2014). A wearable proprioceptive stabilizer (Equistasi^®^) for rehabilitation of postural instability in Parkinson’s disease: A phase II randomized double-blind, double-dummy, controlled study. PLoS ONE.

[69] Serio F., Minosa C., De Luca M., Conte P., Albani G., Peppe A. (2019). Focal vibration training (Equistasi^®^) to Improve posture stability. A retrospective study in Parkinson’s disease. Sensors.

[70] Turbanski S., Haas C.T., Schmidtbleicher D., Friedrich A., Duisberg P. (2005). Effects of random whole-body vibration on postural control in Parkinson’s disease. Res. Sports Med..

[71] Kaut O., Allert N., Coch C., Paus S., Grzeska A., Minnerop M., Wüllner U. (2011). Stochastic resonance therapy in Parkinson’s disease. NeuroRehabilitation.

[72] Kapur S.S., Stebbins G.T., Goetz C.G. (2012). Vibration therapy for Parkinson’s disease: Charcot’s studies revisited. J. Park. Dis..

[73] Haas C.T., Buhlmann A., Turbanski S., Schmidtbleicher D. (2006). Proprioceptive and sensorimotor performance in Parkinson’s disease. Res. Sports Med..

[74] Dincher A. (2021). Effects of Whole Body Vibration on reaction time in Parkinson’s Disease Patients—A pilot study. Neurodegener. Dis. Curr. Res.

[75] Dincher A., Becker P., Wydra G. (2021). Effect of whole-body vibration on freezing and flexibility in Parkinson’s disease—A pilot study. Neurol. Sci..

[76] Dincher A., Wydra G. (2021). Effect of Whole Body Vibration on Balance in Parkinson’s disease—A Randomized Controlled Pilot Study. Alzheimer’s Disease & Treatment.

[77] Corbianco S., Cavallini G., Baldereschi G., Carboncini M.C., Fiamingo F.L., Bongioanni P., Dini M. (2018). Whole body vibration and treadmill training in Parkinson’s disease rehabilitation: Effects on energy cost and recovery phases. Neurol. Sci..

[78] Kaut O., Brenig D., Marek M., Allert N., Wüllner U. (2016). Postural stability in Parkinson’s disease patients is improved after stochastic resonance therapy. Parkinson’s Disease.

[79] Li K.-Y., Cho Y.-J., Chen R.-S. (2021). The Effect of Whole—Body Vibration on Proprioception and Motor Function for Individuals with Moderate Parkinson Disease: A Single—Blind Randomized Controlled Trial. Occup. Ther. Int..

[80] Guadarrama-Molina E., Barrón-Gámez C.E., Estrada-Bellmann I., Meléndez-Flores J.D., Ramírez-Castañeda P., Hernández-Suárez R.M.G., Menchaca-Pérez M., Salas-Fraire O. (2021). Comparison of the effect of whole-body vibration therapy versus conventional therapy on functional balance of patients with Parkinson’s disease: Adding a mixed group. Acta Neurol. Belg..

[81] Pretzer-Aboff I., Elswick R., Gouelle A., Helm N., Blackwell G., Cloud L. (2024). Determination of optimal vibration dose to treat Parkinson’s disease gait symptoms: A clinical trial. Clin. Park. Relat. Disord..

[82] Karbowniczek A., Niewiadomski W., Gasiorowska A., Strasz A., Cybulski G., Palasz E., Niewiadomska G. (2016). Impact of the whole body vibration training on the motor symptoms in Parkinson Disease patients. Park. Relat. Disord..

[83] Karbowniczek A., Niewiadomski W., Gasiorowska A., Strasz A., Cybulski G., Palasz E., Niewiadomska G. (2016). Impact of the whole body vibration training on activity of daily living and quality of life in Parkinson Disease patients. Park. Relat. Disord..

[84] Niewiadomski W., Strasz A., Karbowniczek A., Gasiorowska A., Zylinski M., Pariaszewska K., Cybulski G., Palasz E., Niewiadomska G. (2016). Changes in maximum static force of knee extensors caused by prolonged whole body vibration training in Parkinson Disease patients. Park. Relat. Disord..

[85] Arias P., Chouza M., Vivas J., Cudeiro J. (2009). Effect of whole body vibration in Parkinson’s disease: A controlled study. Mov. Disord..

[86] Ebersbach G., Edler D., Kaufhold O., Wissel J. (2008). Whole body vibration versus conventional physiotherapy to improve balance and gait in Parkinson’s disease. Arch. Phys. Med. Rehabil..

[87] Gaßner H., Janzen A., Schwirtz A., Jansen P. (2014). Random whole body vibration over 5 weeks leads to effects similar to placebo: A controlled study in Parkinson’s disease. Park. Dis..

[88] King L.K., Almeida Q.J., Ahonen H. (2009). Short-term effects of vibration therapy on motor impairments in Parkinson’s disease. NeuroRehabilitation.

[89] Mosabbir A., Almeida Q.J., Ahonen H. (2020). The effects of long-term 40-Hz physioacoustic vibrations on motor impairments in Parkinson’s disease: A double-blinded randomized control trial. Healthcare.

[90] Phuenpathom W., Panyakaew P., Vateekul P., Surangsrirat D., Bhidayasiri R. (2024). Residual effects of combined vibratory and plantar stimulation while seated influences plantar pressure and spatiotemporal gait measures in individuals with Parkinson’s disease exhibiting freezing of gait. Front. Aging Neurosci..

[91] Phuenpathom W., Panyakaew P., Vateekul P., Surangsrirat D., Hiransuthikul A., Bhidayasiri R. (2022). Vibratory and plantar pressure stimulation: Steps to improve freezing of gait in Parkinson’s disease. Park. Relat. Disord..

[92] Romanato M., Guiotto A., Spolaor F., Bakdounes L., Baldassarre G., Cucca A., Peppe A., Volpe D., Sawacha Z. (2021). Changes of biomechanics induced by Equistasi^®^ in Parkinson’s disease: Coupling between balance and lower limb joints kinematics. Med. Biol. Eng. Comput..

[93] Spolaor F., Romanato M., Annamaria G., Peppe A., Bakdounes L., To D.-K., Volpe D., Sawacha Z. (2021). Relationship between muscular activity and postural control changes after proprioceptive focal stimulation (Equistasi^®^) in middle-moderate Parkinson’s disease patients: An explorative study. Sensors.

[94] Yamagami T., Rivera M., Trueblood P., Gomez S. (2018). Effects of Whole Body Vibration Versus Agility Training on Gait Parameters in Individuals with Parkinson’s Disease: A Pilot Study.

[95] Oranges F.P., Greco F., Tarsitano M.G., Quinzi F., Quattrone A., Quattrone A., Emerenziani G.P. (2025). Acute Effects of Whole-Body Vibration on Gait Kinematics in Individuals with Parkinson’s Disease. Appl. Sci..

[96] Peterson D.S., King L.A., Cohen R.G., Horak F.B. (2016). Cognitive contributions to freezing of gait in Parkinson disease: Implications for physical rehabilitation. Phys. Ther..

[97] Scholl J.L., Espinoza A.I., Rai W., Leedom M., Baugh L.A., Berg-Poppe P., Singh A. (2021). Relationships between freezing of gait severity and cognitive deficits in Parkinson’s disease. Brain Sci..

[98] Chow R., Tripp B.P., Rzondzinski D., Almeida Q.J. (2021). Investigating therapies for freezing of gait targeting the cognitive, limbic, and sensorimotor domains. Neurorehabilit. Neural Repair.

[99] Yin S., Liu Y., Zhong Y., Zhu F. (2024). Effects of whole-body vibration on bone mineral density in postmenopausal women: An overview of systematic reviews. BMC Women’s Health.

[100] Liu Y., Fan Y., Chen X. (2022). Effects of whole-body vibration training with different body positions and amplitudes on lower limb muscle activity in middle-aged and older women. Dose-Response.

[101] Hass C.J., Bishop M., Moscovich M., Stegemöller E.L., Skinner J., Malaty I.A., Shukla A.W., McFarland N., Okun M.S. (2014). Defining the clinically meaningful difference in gait speed in persons with Parkinson disease. J. Neurol. Phys. Ther..

[102] Baudendistel S.T., Haussler A.M., Rawson K.S., Earhart G.M. (2024). Minimal clinically important differences of spatiotemporal gait variables in Parkinson disease. Gait Posture.

[103] Taghizadeh G., Eissazade N., Fereshtehnejad S.-M., Taghavi Azar Sharabiani P., Shati M., Mortazavi S.S., Habibi S.A.H., SalemiJuybari M., Mehdizadeh M. (2025). Minimal clinically important difference and substantial clinical benefits for single-and dual-task timed up and go test following motor-cognitive training in Parkinson’s disease. Age Ageing.

